# A Systematic Study on High-Yield Carbon Nanotube Synthesis over TiO_2_-Supported Catalysts: Effect of TiO_2_ Polymorphism and Support Particle Size

**DOI:** 10.3390/nano16191203

**Published:** 2026-09-23

**Authors:** Fernanda Olivares, Sergio Sauceda, Sheila Lascano, Cristina Arévalo, Rodrigo Segura, Marcos Flores, Carolina Parra

**Affiliations:** 1 Departamento de Física, Universidad Técnica Federico Santa María, Av. España 1680, Valparaíso 2390123, Chile; 2 Escuela de Ingeniería Mecánica, Pontificia Universidad Católica de Valparaíso, Los Carrera 01567, Quilpué 2460751, Chile; sergio.sauceda@pucv.cl; 3 Departamento de Ingeniería Mecánica, Universidad Técnica Federico Santa María, Avda. Vicuña Mackenna 3939, Santiago 8940897, Chile; sheila.lascano@usm.cl; 4 Departamento de Ingeniería y Ciencia de los Materiales y del Transporte, Escuela Técnica Superior de Ingeniería, Universidad de Sevilla, Camino de los Descubrimientos s/n, 41092 Sevilla, Spain; carevalo@us.es; 5 Instituto de Química, Universidad de Valparaíso, Av. Gran Bretaña 1111, Valparaíso 2340000, Chile; rodrigo.segura@uv.cl; 6 Department of Physics, Faculty of Physical and Mathematical Sciences, Universidad de Chile, Santiago 8370448, Chile; mflorescarra@uchile.cl; 7 Departamento de Ingeniería Mecánica, Universidad Técnica Federico Santa María, Av. España 1680, Valparaíso 2390123, Chile

**Keywords:** multi-walled carbon nanotube, titanium dioxide nanoparticles, CVD, synthesis optimization

## Abstract

A systematic comparative study of the performance of multi-walled carbon nanotube (MWCNT) synthesis via chemical vapor deposition (CVD) was conducted. Special emphasis is placed on the role of TiO_2_ size and polymorphism as a catalyst support and how these influence the dispersion of the metal nanoparticles and their catalytic behavior. We present three distinct catalytic systems, supported on rutile particles, anatase particles, and anatase nanoparticles, each supporting dispersed Fe–Co nanoparticles anchored to their surfaces. In parallel, catalyst calcination and synthesis parameters, specifically temperature and reaction time, were systematically optimized to maximize carbon fixation yield. The maximum carbon fixation yields were 387% for Rutile/Fe–Co/MWCNT, 321% for Anatase/Fe–Co/MWCNT, and 673% for nanocrystalline Anatase/Fe–Co/MWCNT. These results indicate that the size of the TiO_2_ particles used as a catalyst support, the crystalline phase, and the experimental design for optimizing synthesis parameters play a crucial role in the yield of carbon nanotube synthesis. This suggests that a simple impregnation method can be used to fabricate a TiO_2_-supported metal catalyst and produce MWCNTs with high yield via CVD. This study reveals that catalyst engineering and design will pave the way for the synthesis of nanomaterials tailored to specific applications. The results position nanocrystalline TiO_2_ as a high-performance catalyst support under the experimental design used in this study for the MWCNT synthesis.

## 1. Introduction

Carbon nanotubes (CNTs) have attracted sustained scientific and technological interest due to their exceptional electrical, thermal, mechanical, and chemical properties [1,2,3]. These characteristics make them key components for advanced functional materials in a wide range of applications, including energy systems [4,5,6,7], catalysis [8], aerospace [9] and biomedicine [10]. Compounds based on CNTs and oxides [11,12,13] have emerged as versatile platforms that combine the high electrical conductivity and large specific surface area of CNTs with the chemical stability, catalytic activity, and semiconducting properties of metal oxides. Among these systems, CNT/TiO_2_ composites are especially attractive for applications in photocatalysis [14,15,16,17] and environmental remediation [17,18] as the integration of CNTs with TiO_2_ improves light absorption and promotes efficient electron–hole pair separation, thereby improving pollutant degradation performance. In sensing applications [19,20] this combination provides high conductivity, large surface area, and abundant active sites for interaction with analytes, resulting in enhanced sensitivity and response. Furthermore, CNT/TiO_2_ composites in energy storage systems such as batteries [21,22,23] exhibit higher electrical conductivity, improved ion transport, and reduced structural degradation, leading to higher specific capacity and cycling stability. In the context of biomedicine and bioengineering [24,25,26,27] the synergistic combination of these materials has demonstrated improved antibacterial properties and platforms for biochemical detection, supporting their use in diagnostic and therapeutic applications. It is important to note that in most reported studies, CNT/TiO_2_ composites are produced via physical methods that often result in weak interfacial interactions between both components [25,28,29,30] in contrast to chemical techniques.

The physicochemical properties of CNTs can vary significantly depending on their morphological characteristics [2]. For example, the nanotube diameter directly influences the electronic structure, band gap, and mechanical rigidity [31,32], while the number of walls (single-, double-, and multi-walled CNTs) determines distinct electrical transport behavior and chemical stability [33]. Although multi-walled CNTs (MWCNTs) generally exhibit enhanced mechanical stability, their conductivity differs from that of single-walled CNTs (SWCNTs). Additionally, crystallinity and defect density play a crucial role in determining mechanical strength as well as electrical and thermal conductivity, with higher crystallinity typically associated with better performance [34]. Purity of CNTs is another critical factor, particularly for electronic behavior [35] where minimal metallic impurities and amorphous carbon are desired. These morphological and physicochemical properties are governed by the synthesis method and the nature of the catalytic system employed [36]. Among the available synthesis techniques, chemical vapor deposition (CVD) is widely considered the most scalable and versatile method for CNT production [37], as it enables relatively precise control over nanotube morphology through careful adjustment of catalyst composition, support characteristics, and processing parameters. Transition metals such as Fe, Co, and Ni are the most employed active phases due to their high carbon solubility and catalytic efficiency for hydrocarbon decomposition [38]. Bimetallic catalysts, especially Fe–Co systems, have demonstrated superior performance compared to monometallic counterparts [39], often resulting in higher CNT growth rates, improved control over nanotube diameter, and greater catalytic stability during synthesis.

The catalyst support plays a critical role in catalytic performance, as it governs the dispersion, thermal stability, and sintering resistance of the active metal nanoparticles [40]. Oxide supports such as Al_2_O_3_, MgO, and SiO_2_ have been extensively investigated due to their ability to anchor metal nanoparticles and inhibit coalescence at elevated temperatures [41]. In particular, Al_2_O_3_-supported systems have been widely studied and shown to achieve high CNT yields [39,42], where the physicochemical properties of the Al_2_O_3_ substrate strongly influence the size and distribution of the metal particles, thereby controlling the number of CNT layers, purity, and surface area. Beyond alumina-based systems, TiO_2_ has emerged as a promising support material due to its polymorphism, surface chemistry, and strong metal–support interactions [43]. The two most common crystalline phases of TiO_2_, anatase and rutile, differ significantly in crystal structure, surface energy, and reducibility [44], which can notably influence catalyst behavior and CNT growth mechanisms. Furthermore, the particle size of TiO_2_, ranging from micrometer-scale powders to nanometer-sized particles, introduces additional variables that affect metal dispersion, catalyst activation, and carbon diffusion kinetics.

Despite the growing body of research on CNT growth over oxide-supported catalysts, studies specifically addressing TiO_2_-supported systems remain relatively limited [45] and many report low CNT growth yields or carbon fixation [46]. Moreover, systematic investigations that simultaneously evaluate the effects of the TiO_2_ crystalline phase, particle size, catalyst calcination temperature, and CNT synthesis parameters are scarce. Understanding how anatase-supported Fe–Co and rutile TiO_2_ catalysts respond to changes in catalyst-preparation temperature and CNT growth conditions, such as synthesis temperature and residence time, remains limited. This knowledge gap hinders the rational design of catalytic systems capable of producing CNTs with customized structural characteristics and high yield.

From a theoretical perspective, CNT growth during CVD processes is commonly described by vapor–liquid–solid (VLS) or vapor–solid–solid (VSS) mechanisms, in which the size, composition, and phase of the catalyst nanoparticle govern carbon solubility, precipitation rate, and nanotube diameter. Temperature plays a dual role by influencing both catalyst nanoparticle evolution (e.g., reduction, alloying, and sintering) and the kinetics of hydrocarbon decomposition and carbon diffusion. Similarly, synthesis time affects not only the overall CNT yield but also the defect density, nanotube length, and catalyst deactivation. Therefore, a systematic experimental framework that can decouple and quantify the effects of these parameters (temperature and time) is required to establish reliable structure–process–property relationships.

The present work investigates the synthesis of MWCNTs on Fe–Co catalysts supported on three TiO_2_ systems: submicrometric rutile, submicrometric anatase, and nanocrystalline anatase. The three supports were selected to allow two independent comparisons within the same dataset. The first comparison, between submicrometric rutile and submicrometric anatase (two powders whose average particle sizes are within the same order of magnitude), primarily analyzes the effect of the crystalline phase. The second comparison, between submicrometric and nanocrystalline anatase (two powders with identical phase and identical calcination temperature), analyzes the effect of the support particle size and associated surface area. The study emphasizes catalyst calcination temperature, CNT synthesis temperature, and growth time, which were optimized using a statistically designed experimental approach that quantifies the importance of each factor and their interactions. Structural, morphological, and thermal characterization was performed using FESEM, HRTEM, XRD, Raman spectroscopy, and thermogravimetric analysis to correlate catalyst properties with the growth behavior of CNTs. The novelty of this work lies not in the individual use of TiO_2_ as a support, which has been previously described, but in the combination of three elements not previously brought together for this system: a replicated factorial design that quantifies the operating range of each support rather than a single optimum point; the deliberate separation of phase and particle-size contributions; and carbon fixation yields approaching 700%, more than an order of magnitude higher than previously reported values for TiO_2_-supported catalysts.

## 2. Materials and Methods

### 2.1. Design of Experiments

To statistically evaluate the effect of MWCNT production parameters, the response variable was defined as the carbon fixation yield (Y_C_), with the objective of maximizing its value. In the first experimental design, the catalyst calcination temperature (400, 450, 500, 550, 600, and 650 °C) was considered a continuous and controllable variable. After identifying the optimal calcination temperature for each catalyst, a second experimental design was used to investigate the effect of CNT synthesis conditions. In this stage, synthesis temperature (670, 700, and 730 °C) and the residence time (30, 45, and 60 min) were selected as independent variables. A 3 × 3 multilevel factorial design was performed in three independent replicate blocks, with the nine experimental conditions randomized within each block, resulting in a total of 27 experiments and allowing the repeatability of the process to be evaluated across blocks.

For each catalytic system, a second-order polynomial model was initially fitted using coded levels (−1, 0, +1) for synthesis temperature (A) and time (B). In the model, β_0_ represents the intercept, β_1_ and β_2_ are the linear effects of temperature and time, respectively, β_12_ is the temperature–time interaction effect, and β_11_ and β_22_ are the quadratic effects of temperature and time. B_k_ represents the effect of the kth replicate block, accounting for variability among the independent experimental blocks, while ε represents the random experimental error not explained by the model. Non-significant higher-order terms (*p* > 0.05) were removed while preserving model hierarchy, and the block effect was retained in the analysis.

(1) $$\%Y=\beta_{0}+\beta_{1}A+\beta_{2}B+\beta_{11}A^{2}+\beta_{22}B^{2}+\beta_{12}AB+B_{k}+\varepsilon$$

Statistical analysis was performed using Statgraphics^®^ 18 software. The full matrix of the first experimental design, comprising the six calcination temperatures evaluated for each of the three supports under fixed CNT growth conditions (730 °C, 30 min), is provided in Table S1. The factor levels of the second design, together with the optimal calcination temperature adopted for each catalyst (500 °C for rutile/Fe–Co and 600 °C for both anatase/Fe–Co and n-anatase/Fe–Co), are summarized in Table S2. The complete set of 27 runs per catalyst—including the initial catalyst mass, the mass recovered after growth, and the resulting carbon fixation yield with its standard deviation—is reported in Tables S3–S5, and the corresponding analyses of variance are compiled in Tables S6–S8. Reporting the three data sets in full allows the response surfaces and Pareto charts discussed in Section 3.2 to be reproduced independently, and makes explicit the contrast in operating window between the rutile-supported and the anatase-supported systems.

### 2.2. Catalyst Preparation

The catalyst consisted of Fe–Co nanoparticles supported on TiO_2_ particles and nanoparticles (TiO_2_-Fe_2_O_3_-Co_3_O_4_) with a nominal composition ratio of 5:2:6 by weight, respectively. Cobalt (II) acetate tetrahydrate (98% Alfa aesar) was first dissolved in 100 mL of 2-propanol (99.5% J.T. Baker) under magnetic stirring for 5 min. Subsequently, iron (II) oxalate dihydrate (99% Alfa aesar) was added and the mixture was stirred for an additional 5 min. TiO_2_ particles were then incorporated into the solution, followed by sonication using a probe sonicator for 30 min to ensure homogeneous dispersion. The resulting suspension was dried at 60 °C for 24 h and subsequently at 130 °C for an additional 24 h. The dried material was ground using an agate mortar and then calcined in air for 4 h at temperatures ranging from 400 °C to 650 °C. Calcination was carried out in an oxidizing atmosphere of air to promote the formation of metal oxide nanoparticles.

### 2.3. Carbon Nanotube Synthesis

CNTs were synthesized by chemical vapor deposition (CVD) (Across International TFM2-1200, Livingston, NJ, USA) at atmospheric pressure in a horizontal tube furnace. The catalyst was spread onto a 10 cm long quartz container, positioned in the center of a 10 cm diameter, 100 cm long quartz tube inside the furnace. Prior to heating, the system was purged with argon (Ar) 99.999% at a rate of 300 sccm for 5 min to remove residual air. The furnace was then heated in a temperature range of 670–730 °C at a rate of 50 °C/min under a constant Ar flow (300 sccm). Once the target temperature was reached, CNT growth was initiated by introducing ethylene C_2_H_4_ (100 sccm) and hydrogen (30 sccm) 99.999%, while maintaining the Ar flow. The synthesis time varied between 30 and 60 min. At the end of the synthesis, the furnace was turned off, and the ethylene and hydrogen flows were stopped. The system was allowed to cool to room temperature under continuous Ar flow. Performance was evaluated using the following equation:

(2) $$Y_{C}(\%)=\frac{{(m}_{f}-m_{i})}{m_{i}}\;\times 100\;\;\;\;$$

where Y_C_ is the carbon fixation yield, m_f_ is the final recovered mass after the synthesis is completed (catalyst + fixed carbon), and m_i_ is the initial mass of the catalyst, previously dried and calcined before synthesis. Therefore, the mass increase during synthesis is assumed to result exclusively from carbon deposition due to the growth of MWCNTs and other carbonaceous species via C_2_H_4_ decomposition.

The initial catalyst mass was kept at 0.1000 ± 0.0010 g in every run, as documented in Tables S3–S5, so that the differences in carbon fixation yield reported in Section 3.2 can be attributed exclusively to the nature of the support and to the growth conditions, and not to variations in the amount of catalyst loaded.

### 2.4. Characterization of Catalyst and CNT

Morphological characterization was performed using a field emission scanning electron microscope (FESEM, Thermo Scientific Quattro S, Brno, Czech Republic) equipped with a secondary electron detector. High-resolution transmission electron microscopy (HRTEM) analysis was performed using a Thermo Fisher Talos F200C microscope, Brno, Czech Republic. Statistical analysis of particle, nanoparticle, and nanotube diameters was performed using FESEM and HRTEM digital micrographs and ImageJ software 1.54d. Raman spectroscopy measurements were carried out on a Renishaw inVia with a 532 nm laser source (Renishaw, New Mills, UK). The thermal stability of the synthesized MWCNT was examined by thermogravimetric analysis (TGA) using a Mettler Toledo TGA/DSC 850 instrument (Mettler Toledo, Greifensee, Switzerland). Samples were heated at a rate of 10 °C/min up to 1000 °C under a synthetic air atmosphere. The crystalline structure of the samples was analyzed by X-ray diffraction (XRD) using a Bruker D8 Discover A25 diffractometer (Billerica, MA, USA) equipped with Cu Kα radiation and a graphite monochromator. The diffraction patterns were recorded over a 2θ range of 20–80° at a scan rate of 0.02°·s^−1^. These same acquisition conditions were applied to the complete calcination series of each catalyst (400–650 °C), whose diffraction patterns are compiled in Figures S1–S3 and used in Section 3.1 to rationalize the phase-dependent optimum calcination temperature. X-ray photoelectron spectroscopy (XPS) was performed to evaluate the chemical states of the surface atomic species. Measurements were carried out using a SPECS XPS spectrometer (SPECS Surface Nano Analysis GmbH, Berlin, Germany) equipped with a PHOIBOS 150 hemispherical electron energy analyzer and a 1D-DLD detector. Unfiltered Al Kα radiation (hν = 1486.6 eV) was generated using an aluminum anode. The base pressure of the main analytical chamber was maintained at approximately10^−7^ Pa during data acquisition. Surface atomic ratios were calculated by integrating core-level peak areas normalized by the acquisition parameters, manufacturer-provided atomic sensitivity factors (ASFs), and transmission functions.

## 3. Results and Discussion

### 3.1. Preparation of the TiO_2_-Fe–Co Catalyst

Figure 1 presents the morphological and vibrational characterization of TiO_2_ particles in the rutile and submicrometric and nanometric anatase phases, providing important information on the effect of the crystalline phase and particle size on the physicochemical properties of the materials.

FESEM micrographs of rutile particles (Figure 1a) show irregularly shaped agglomerates composed of relatively large primary particles with a quasi-spherical geometry. This morphology is consistent with the thermodynamically stable nature of the rutile phase, which typically forms larger crystallites through crystal growth. Anatase particles (Figure 1b) exhibit a similar quasi-spherical morphology, characterized by smaller particle sizes and a high aggregation state, reflecting the metastable nature of anatase and its tendency to form agglomerates. The HRTEM image of the anatase nanoparticles (Figure 1c) provides structural detail, showing a quasi-spherical geometry and a high aggregation state, while still clearly distinguishing the boundaries of each nanoparticle.

Particle-size distributions, derived from statistical analysis of microscopy images, are shown in Figure 1d–f. The number of particles measured for each phase was N = 68 for submicrometric rutile, N = 82 for submicrometric anatase, and N = 106 for nanometric anatase. Rutile particles (Figure 1d) exhibit a broad size distribution centered on particle diameters of 238.7 ± 71.4 nm, which are larger than those of anatase particles, consistent with FESEM observations. Anatase particles (Figure 1e) also exhibit a broad size distribution with a smaller mean particle size of 188.6 ± 71.4 nm. Anatase nanoparticles (Figure 1f), by contrast, have a remarkably reduced mean size of 30.2 ± 6.7 nm with a relatively narrow distribution. This size reduction is expected to increase the specific surface area, a key parameter for catalytic performance and metal dispersion in supported catalytic systems.

The Raman spectra of rutile particles (Figure 1g), anatase particles (Figure 1h), and anatase nanoparticles (Figure 1i) corroborate the chosen crystalline phases. The rutile spectrum is characterized by the presence of its characteristic Raman modes at ~239, 450, and 610 cm^−1^, corresponding to multiple phonon scattering, E_g_, and A_1g_ vibrational modes, confirming the formation of the rutile phase without detectable secondary phases [44,47]. The anatase particles exhibit the typical bands at ~142, 396, 514, and 641 cm^−1^, corresponding to the E_g_, B_1g_, A_1g_, and E_g_ vibrational modes [44,47], respectively, with sharper and more intense peaks compared to rutile. The Raman spectrum of anatase nanoparticles exhibits the same peaks as anatase particles, but a broadening in the Eg mode is observed, which can be attributed to phonon confinement effects resulting from crystal size reduction and increased surface-to-volume ratio [44,48]. These spectral changes indicate alterations in lattice dynamics, associated with modified Ti-O bonding environments, surface-induced strain, and increased anharmonicity in the anatase nanocrystalline structure. These spectral features provide strong evidence for crystal size reduction and increased surface-to-volume ratio, factors that influence charge transport, surface reactivity, and metal–support interactions in catalytic systems.

Subsequently, an optimization study of the calcination temperature of the rutile/Fe–Co (R), anatase/Fe–Co (A), and n-anatase/Fe–Co (n-A) catalysts was conducted to analyze their impact on carbon fixation yield. Table S1 summarizes the calcination temperatures investigated for the TiO_2_/Fe–Co catalysts with the aim of optimizing carbon fixation yield during CNT synthesis. Calcination temperature is a critical parameter, as it can influence a crystallographic phase change in the TiO_2_ support, the dispersion and oxidation state of the active Fe–Co species, and the strength of the metal–support interactions that govern catalytic activity during CNT growth. The CNT synthesis temperature and time parameters (730 °C and 30 min, respectively) were kept constant to observe the effect of the catalyst calcination temperature.

Figure 2a shows the evolution of carbon fixation yield with calcination temperature for the three catalysts. Each system exhibits a well-defined optimum, but at a different temperature: the rutile-supported catalyst reaches its maximum yield after calcination at 500 °C, while both anatase-supported catalysts do so at 600 °C. Low calcination temperatures (400–450 °C) result in suboptimal yields, while high calcination temperatures (650 °C) lead to a decrease in yield that can be attributed to sintering of the Fe–Co particles and partial loss of the active surface. Under these fixed growth conditions, the rutile- and n-anatase-supported catalysts exhibit comparable maximum yields (353 ± 13% and 334 ± 5%, respectively), both higher than that of the submicrometer anatase system (322 ± 3%).

Because the analysis in Figure 2a covers the entire 400–650 °C range for all three catalysts under a common set of growth conditions, it also constitutes constant-calcination temperature control. At a common calcination temperature of 600 °C, with growth fixed at 730 °C for 30 min, the yields are 321 ± 15% for rutile/Fe–Co, 229 ± 23% for anatase/Fe–Co, and 334 ± 5% for n-anatase/Fe–Co; at a common calcination temperature of 500 °C, the corresponding values are 353 ± 13%, 209 ± 7%, and 310 ± 15%. Therefore, the ranking of the two anatase systems is preserved under a common thermal history. When observing the temperature sweep, the submicrometric and nanometer anatase catalysts show the same peaks and valleys, but the latter consistently exhibits higher performance. Rutile, on the other hand, shows a different thermal history with similar yield percentages to the nanometer anatase catalyst under this set of parameters. The complete numerical dataset is provided in Table S1.

Figure 2b presents the XRD patterns of the TiO_2_/Fe–Co catalysts calcined under their respective optimal conditions: rutile/Fe–Co calcined at 500 °C (blue), anatase/Fe–Co calcined at 600 °C (red), and n-anatase/Fe–Co calcined at 600 °C (black). The diffraction patterns confirm predominant crystallization of TiO_2_ into the tetragonal phases anatase (PDF 00-021-1272) and rutile (PDF 00-021-1276), as shown by the indexed major reflections. Minor amounts of cubic cobalt oxide (PDF 00-042-1467) and smaller quantities of iron oxide (PDF 04-015-9120) were also detected.

FESEM images of the optimized catalysts are shown in Figure 2c–h. Figure 2c–e show images at lower magnification, and Figure 2f–h at higher magnification, respectively. The morphology of the rutile/Fe–Co catalyst calcined at 500 °C (Figure 2c) exhibits relatively similar agglomerations compared to the anatase/Fe–Co catalyst (Figure 2d) and a coral-like structure. However, analysis of the same images at higher magnification (Figure 2f,g) reveals that the surface of the rutile catalyst is rougher than that of the anatase catalyst, suggesting a larger surface area and, therefore, greater exposure of the catalyst particles. This could favor CNT growth and is consistent with the slightly higher carbon fixation yield. Conversely, the anatase/Fe–Co catalyst calcined at 600 °C (Figure 2d,f) exhibits more compact agglomerates compared to the other two catalysts, which may translate to a smaller surface area and fewer active sites for CNT growth. This is consistent with the anatase catalyst having the lowest carbon fixation yield of the three catalysts studied. The n-anatase/Fe–Co catalyst calcined at 600 °C (Figure 2e,h) has a low-density morphology, with agglomerates that appear much larger at low magnification. However, at higher magnification, it shows a more open, less compact surface architecture than the other two catalysts, with higher roughness. This morphology maximizes active-site availability and facilitates carbon diffusion and precipitation during CNT synthesis. This analysis agrees with the much higher carbon fixation yield obtained using this catalyst.

Overall, these results indicate that optimizing the calcination temperature is crucial for maximizing carbon fixation yield in CNT synthesis over TiO_2_/Fe–Co catalysts. The results show that rutile-based supports and nanocrystalline anatase outperform submicrometric anatase. This may be due to their ability to stabilize finely dispersed Fe–Co nanoparticles and promote favorable metal–support interactions. These findings establish a direct correlation between calcination conditions, catalyst structure, and carbon fixation yield, providing a framework for rationally designing high-performance CNT synthesis catalysts.

It should be stressed that the ranking obtained at this stage reflects the catalyst’s thermal history under one set of growth conditions, not the ultimate potential of each support. This is precisely the reason for the two-stage design adopted here: the calcination temperature is a catalyst-preparation variable that must be optimized for each support before the growth window of the different supports can be compared meaningfully, since comparing all three systems at a single arbitrary calcination temperature would confound the intrinsic properties of the support with a thermal history that is optimal for only one of them. Once the growth parameters are themselves optimized, as shown in Section 3.2, the n-anatase system moves well ahead of the other two.

### 3.2. Optimization of CNT Synthesis Through Experimental Design

Table S2 summarizes the synthesis parameters evaluated to optimize carbon fixation yield during CNT growth on TiO_2_/Fe–Co catalysts, specifically the CNT synthesis temperature and time. These parameters play a fundamental role in determining the hydrocarbon decomposition kinetics and carbon diffusion through the active-metal phase, which regulate CNT formation. The experimental design shows that both parameters significantly influence carbon fixation yield, although their relative impact varies with the crystalline phase and particle size of the TiO_2_ support. This behavior highlights the importance of adapting the synthesis conditions to the physicochemical characteristics of each catalytic system.

Figure 3a–c present the response surface plots obtained, illustrating the combined effect of temperature and synthesis time on carbon fixation yield for the three catalysts. For rutile/Fe–Co/CNT (Figure 3a), the response surface exhibits a relatively narrow optimum region, indicating limited operational flexibility. The maximum yield of 387% is achieved with 60 min and 730 °C of synthesis, while the minimum yield of 337% is obtained with 30 min and 670 °C. This means that despite varying both parameters, the yield increase is small. In contrast, for anatase/Fe–Co/CNT (Figure 3b), a significant increase is observed between the minimum yield of 143% at 30 min and 670 °C and the maximum yield of 321% at 60 min and 730 °C. Although the maximum carbon fixation yield is comparable for CNTs grown on rutile and anatase, the minimum yield is significantly lower for the anatase-based catalyst. This is directly related to the FESEM study (Figure 2c,d), which observed that the morphology of the catalyst supported on anatase is more compact than that supported on rutile. This translates to a smaller surface area and, therefore, fewer active sites for forming catalytic metal particles.

On the other hand, the n-anatase/Fe–Co/CNT (Figure 3c) exhibits the best expected yield. The minimum yield is 334% at 30 min and 730 °C, and the maximum is 673% at 60 min and 670 °C, evidencing a nonlinear dependence of carbon fixation yield on synthesis time and temperature.

Consequently, the particle size reduction of TiO_2_ anatase favorably modulates interfacial chemistry with Fe–Co, suggesting an increased surface area and density of anchoring and nucleation sites, and improved dispersion and stabilization of metallic nanoparticles, leading to more catalytically active diameters [49].

The ANOVA results for the three catalytic systems are graphically represented by the Pareto diagrams in Figure 3d–f, while the corresponding numerical outputs are compiled in Tables S6–S8. Statistical significance was established at the 95% confidence level (*p* < 0.05).

The fitted regression equations for the three catalytic systems are given below, where %Y is the carbon fixation yield, T is the synthesis temperature (°C), and t is the synthesis time (min).

Anatase/Fe–Co/CNT:

(3) %Y=−282.118+0.549T−13.879t+0.024Tt

n-Anatase/Fe–Co/CNT:

(4) $$\%Y=65639.100-184.920T+23.287t+0.130T^{2}-0.209t^{2}$$

Rutile/Fe–Co/CNT:

(5) %Y=152.708+0.226T+1.189t

For Rutile/Fe–Co/CNT (Figure 3d, Table S6), only synthesis time reached statistical significance (*p* = 0.0034), while the effect of temperature was not significant (*p* = 0.2261). The block effect was also significant (*p* = 0.0050), indicating non-negligible variability among the three independent replicate blocks. The model yielded an adjusted R^2^_adjust_ of 45.7%, reflecting both the limited sensitivity of carbon fixation yield to the synthesis conditions within the studied range and the variability observed between replicate blocks, consistent with the limited variation across the rutile response surface analyzed earlier. Notably, block variability was comparable in magnitude to that explained by the controlled factors.

For Anatase/Fe–Co/CNT (Figure 3e), both temperature and synthesis time are statistically significant. Table S7 shows that the two main effects are highly significant (*p* < 0.0001) and that their two-factor interaction AB is also significant (*p* = 0.0153), while the block factor is not (*p* = 0.6836); the model accounts for 93.4% of the adjusted variance. This result indicates that CNT growth on anatase-supported catalysts is determined by a more complex interaction between kinetic and thermodynamic factors.

For n-Anatase/Fe–Co/CNT, the carbon fixation yield depends nonlinearly on both synthesis temperature and time, leading to a maximum yield at the lowest temperature tested (670 °C), unlike the monotonic behavior observed for the two submicrometric supports. This curved response is evidenced by the Pareto chart in Figure 3f and by the statistical significance of both main factors and the quadratic terms AA (*p* < 0.0001) and BB (*p* = 0.0178) reported in Table S8. This result indicates that CNT growth on anatase-supported catalysts is governed by a complex interaction between kinetic and thermodynamic factors.

Mechanistically, this reversal suggests a metal–support electronic interaction (EMSI) enhanced by nanocrystalline support: because the Fe–Co particles are already finely dispersed and strongly anchored, additional thermal energy no longer improves activation but instead promotes particle coarsening and premature deactivation, so that the optimum shifts towards milder conditions. The adjusted R^2^ values of 93.4% (anatase, Table S7) and 86.6% (n-anatase, Table S8) confirm the adequacy of these models and the predictive capability of the corresponding response surfaces [50]. The much lower adjusted R^2^ of the rutile model (45.7%, Table S6) should not be read as a failure of the methodology but as a result in its own right: no combination of temperature and time within the domain explored is able to substantially modify the carbon fixation yield of this catalyst, which is precisely the operational limitation that the anatase-based supports overcome.

Figure 3g–i present bar graphs that directly display the experimentally obtained carbon fixation yield at selected synthesis times and temperatures for each catalyst. These results make it easier to observe the trends predicted by response surface analysis. For Rutile/Fe–Co/CNT (Figure 3g), the difference in yield values is not very pronounced. This behavior reflects the limited capacity of the rutile support to stabilize the active sites under prolonged or high-temperature synthesis conditions. The Anatase/Fe–Co/CNT system (Figure 3h) shows consistently higher yield variations across a wide range of synthesis parameters. The highest yield is obtained with n-anatase/Fe–Co/CNT (Figure 3i), making n-anatase the most effective support among the systems studied. The numerical values plotted in Figure 3g–i, together with their standard deviations, are listed in Tables S3–S5.

The analysis shows that CNT temperature and synthesis time are critical parameters controlling carbon fixation yield, and their optimal values depend largely on the phase and morphology of the TiO_2_ support. While rutile-based catalysts exhibit limited operational flexibility, anatase supports, and particularly nanocrystalline anatase supports, allow for greater variations in yield. Furthermore, these results indicate that n-anatase/Fe–Co catalysts offer high-yield carbon fixation in CNTs, thanks to improved metal dispersion. It is also possible that the more reactive nanoparticles result in stronger metal–support interactions and greater resistance to deactivation.

It is worth emphasizing that this comparison is made on the basis of the complete data set of Tables S3–S5 rather than on a single best run: the n-anatase/Fe–Co system exceeds 400% carbon fixation yield in seven of the nine conditions tested and never falls below 334.6%, so that its performance advantage is robust with respect to the choice of operating point and not the result of a narrowly optimized condition.

Placing these values in the context of the literature requires care, because studies on CNT synthesis use different definitions of yield or productivity, different catalyst masses and active-metal loadings, different carbon precursors, and different reaction times, so reported percentages are not directly comparable. TiO_2_ has moreover been used only sparingly as a support for metallic catalysts in CNT synthesis via CVD, which restricts the number of directly relevant reference systems. Some articles report significantly lower yields, such as the study by Mitra et al. (2022) [46], which obtained a maximum of 32% with a 5Co1Mo-TiO_2_ catalyst. Other articles do not even report the yields obtained. For example, Dechakiatkrai et al. (2009) [51] used TiO_2_ (rutile and anatase mixture)/Fe nanoparticles and obtained CNTs with diameters close to 100 nm. On the other hand, the results obtained in this study are comparable to other systems with similar carbon fixation yields but using a support other than TiO_2_, as is the case with Martinez-Vázquez et al. (2026) [52], Fe:SiO_2_ systems were used, achieving a maximum carbon fixation yield of 609%. To make this comparison explicit, Table 1 compiles the TiO_2_-supported and TiO_2_-based systems for which MWCNT growth by CVD has been reported, together with the parameters that determine whether the reported yields can be placed on a common scale.

Two points emerge from Table 1. First, the literature does not use a uniform definition of the response variable. The carbon fixation yield definition adopted here, with the mass of the calcined catalyst (supported TiO2 plus Fe and Co oxides) as the denominator, matches the definition reported by Mintra et al. Secondly, it is not possible to make assertions about carbon fixation performance when comparing the currently available data for TiO_2_-supported systems. Although the same synthesis time (30 min) is used for the reported catalysts and the work of Mitra et al., the carbon source, synthesis temperature, and metals studied differ.

The three catalytic systems compared here differ simultaneously in the crystalline phase of the support, the particle size of the support, and, in the case of the rutile system, the calcination temperature at which the catalyst was prepared. Therefore, any claim attributing the observed differences to only one of these variables would be unfounded, and the conclusions of this work are consequently divided into two distinct comparisons.

The first comparison, between submicrometric rutile and submicrometric anatase, primarily analyzes the effect of the crystalline phase, as the two average particle sizes differ by only 26% and their distributions substantially overlap. However, this is not a precise phase comparison, given that the two catalysts were calcined at different temperatures: 500 °C and 600 °C, respectively. The data support the following: under their respective optimum preparation and growth conditions, both systems achieve similar maximum yields, 387.1 ± 19.7% for rutile and 321.2 ± 1.5% for anatase, but they behave in opposite ways with respect to growth parameters. The rutile system is virtually insensitive to temperature and time, with yields between 337% and 387% across the entire range, whereas the anatase system varies by a factor of 2.2 over the same range and only approaches the rutile yield at the upper end of the design region. In other words, the difference between the two phases in this study lies in the shape of the operating range, rather than in the maximum achievable yield. The constant-calcination temperature comparison in Section 3.2 corroborates this conclusion.

The second comparison, between submicrometric anatase (188.6 ± 71.4 nm) and nanocrystalline anatase (30.2 ± 6.7 nm), is considerably clearer. Both supports have the same crystalline phase and were calcined at the same temperature (600 °C), and the only appreciably different catalyst variable is the support particle size, which is reduced by a factor of six. Between these two systems, the maximum carbon fixation yield increases from 321.2 ± 1.5% to 673.9 ± 31.4%. In addition, at the respective optimum growth conditions, the mean nanotube diameter decreases from 48.4 ± 14.9 nm to 28.8 ± 10.5 nm, and the I_D_/I_G_ ratio decreases from 1.15 to 0.59. Because this comparison keeps the crystalline phase and calcination temperature constant, the particle-size effect is the central result of this work. Within the anatase phase, reducing the support particle size to the nanometer range roughly doubles the maximum carbon fixation yield. However, the smaller diameter and lower defect density of the nanotubes cannot be attributed exclusively to support particle size, because the n-anatase product was grown at 670 °C rather than 730 °C (Section 3.3).

### 3.3. Morphological and Structural Analysis of CNTs Grown over TiO_2_/Fe–Co Catalyst

The samples characterized in this section were grown under the conditions that maximize the experimental carbon fixation yield of each factorial design (Tables S3–S5): 730 °C for 60 min for rutile/Fe–Co and anatase/Fe–Co, and 670 °C for 60 min for n-anatase/Fe–Co. The comparison that follows therefore contrasts each catalytic system at its own optimum, and not under a common but arbitrary set of conditions. This choice implies that the three products were not grown under a common thermal condition: the n-anatase/Fe–Co sample was synthesized 60 °C lower than the other two. Since the CVD temperature alone governs the C_2_H_4_ decomposition kinetics, catalyst growth, carbon supersaturation in the metal particle, and the degree of graphitization, the differences in diameter, defect density, and thermal stability described below should be interpreted as the combined result of the support and growth temperature, and cannot be attributed solely to the TiO_2_ polymorph. Therefore, the purpose of this section is to document the morphology and structural quality of the nanotubes that each catalytic system produces under its optimum condition, and not to isolate the intrinsic effect of the support on the carbon nanotube structure.

Figure 4a–c present FESEM images of CNTs synthesized under optimized conditions for each catalytic system. Clear differences in CNT diameter and size homogeneity are observed among the three catalytic systems under their respective optimal growth conditions. CNTs grown on rutile/Fe–Co at 730 °C for 60 min (Figure 4a) exhibit a relatively heterogeneous morphology, characterized by CNTs with irregular diameters and the presence of intertwined structures. Catalyst clusters are also observed. CNTs synthesized on anatase/Fe–Co under identical conditions (Figure 4b) show clearly larger-diameter CNTs and exhibit regions with anatase catalyst clusters. Smaller and more homogeneous CNT structures are obtained using n-anatase/Fe–Co at a lower synthesis temperature of 670 °C (Figure 4c). The CNTs grown on this catalyst exhibit smaller diameters than those obtained with the other two catalysts, showing that this system can produce well-structured CNTs at a lower synthesis temperature. This narrowing cannot be ascribed to the support alone, since this sample was grown at 670 °C rather than 730 °C.

Figure 4d–f show the diameter histograms of the CNTs and the descriptive statistics for the three catalytic systems. The number of nanotubes measured for each catalyst was N = 150 for rutile/Fe–Co, N = 65 for anatase/Fe–Co, and N = 118 for n-anatase/Fe–Co. The CNTs synthesized on rutile/Fe–Co (Figure 4d) exhibit a wide diameter distribution and a standard deviation of 31.1 ± 10.7, reflecting the heterogeneous size of the active metal nanoparticles. For anatase/Fe–Co (Figure 4e), the diameter distribution and dispersion are also high, with an average diameter of 48.4 ± 14.9, much larger compared to the CNTs grown on rutile. This result suggests that Fe and Co catalyst particles nucleate at fewer reaction sites but accumulate more, generating larger metal particles and, consequently, CNTs with larger diameters than those obtained with the other catalysts. The narrowest diameter distribution is observed for n-anatase/Fe–Co (Figure 4f), where the CNTs have average diameters smaller than 28.8 ± 10.5. This behavior suggests a large surface area and uniform nanocrystalline anatase, which would favor anchoring of the Fe–Co nanoparticles and restrict their growth. However, this sample was grown 60 °C below the other two, and a lower CVD temperature independently limits catalyst coarsening.

Figure 5a–c show the Raman spectra of the CNTs synthesized with the three catalysts. All spectra exhibit the characteristic D band (~1350 cm^−1^), G band (~1580 cm^−1^), and 2D band (~2590 cm^−1^) associated with multi-walled carbon nanotubes (MWCNTs). However, this technique does not rule out the presence of small amounts of amorphous carbon. The intensities of the 2D and G bands are also consistent with the formation of multi-walled nanotubes, where the 2D band is less intense than the G band. Furthermore, the peaks corresponding to the crystalline phases of TiO_2_ are visible in all three spectra (Figure 5a rutile, Figure 5b anatase, and Figure 5c n-anatase).

The CNTs grown on rutile/Fe–Co (Figure 5a) exhibit an I_D_/I_G_ ratio of 0.61 and an I_2D_/I_G_ ratio of 0.88, indicating a moderate degree of structural defects and disordered carbon. This observation is consistent with the heterogeneous morphology and similar diameter distribution of the CNTs grown on n-anatase/Fe–Co, observed in the FESEM analysis. In the case of anatase/Fe–Co (Figure 5b), the I_D_/I_G_ ratio is 1.15, and the I_2D_/I_G_ ratio is 0.57, reflecting lower graphitic ordering and a high number of defects. Nanotubes supported on n-anatase/Fe–Co (Figure 5c) have an I_D_/I_G_ ratio of 0.59 and an I_2D_/I_G_ ratio of 0.89. These latter nanotubes exhibit the lowest I_D_/I_G_ ratio, indicating fewer defects. The I_2D_/I_G_ ratio also indicates higher graphitic order compared to nanotubes supported on submicrometric anatase, but like that of nanotubes supported on rutile, which is consistent given their very similar diameter and morphology. The lowest I_D_/I_G_ ratio of the three systems is thus obtained for nanotubes grown on n-anatase/Fe–Co at 670 °C, showing that this system can produce high-quality carbon nanotubes at a lower synthesis temperature. This observation does not by itself establish that the lower defect density originates from the support, because growth temperature is a well-documented determinant of the degree of graphitization and the relative D-band intensity. The differences in I_D_/I_G_ and I_2D_/I_G_ among the three systems are therefore attributed to the combined effect of the support and of the growth temperature.

Figure 5d presents the TGA/DSC profiles of the carbonaceous products synthesized on the three Fe–Co/TiO_2_ catalytic systems. The rutile-derived material exhibits an earlier and broader mass loss, whereas the anatase- and n-anatase-derived materials retain a larger fraction of their initial mass at higher temperatures. At approximately 1000 °C, the residual masses are 42.6%, 51.3%, and 62.2% for Rutile/Fe–Co/CNT, Anatase/Fe–Co/CNT, and n-Anatase/Fe–Co/CNT, respectively. However, these residual values should not be interpreted directly as the inorganic catalyst fraction or as a measure of CNT purity, since the curves continue to decrease at the upper temperature limit and do not reach a clear final mass plateau indicating higher thermal stability and structural uniformity. The DSC profiles reveal clear differences in the thermal response of the three carbonaceous products. The rutile-derived sample shows an earlier and broader deviation of the heat-flow signal from the baseline, beginning at lower temperatures and extending over a wide temperature range. In comparison, the anatase- and n-anatase-derived samples maintain a more stable thermal response up to higher temperatures, with the n-anatase system showing the most gradual evolution before the high-temperature oxidation region. This behavior is consistent with differences in the thermal stability and structural organization of the deposited carbonaceous species. Localized abrupt changes in the DSC signals coincide with sharp features in the DTG curves provided in Figure S5 of the Supplementary Information. These features may arise from transient instrumental effects or rapid mass changes associated with oxidation reactions.

These results are consistent with Raman and morphological observations. As with the preceding techniques, however, the three products were obtained at different growth temperatures, so the higher thermal stability measured for the n-anatase-derived material reflects the product delivered by that system under its best-performing condition and is not, on its own, evidence of an effect of the support.

Taken together, the results of this section show that the n-anatase/Fe–Co system, operated at its best condition (670 °C, 60 min), simultaneously delivers the highest carbon fixation yield (Table S5), the widest range of conditions over which high yields are accessible (Tables S5 and S8), and the nanotubes with the narrowest diameter distribution and the lowest ID/IG ratio of the three systems. High yield and high quality are therefore not traded off against each other in this system, which is the central practical outcome of this work. Because the structural characterization was performed at the optimum for each system, and therefore at different growth temperatures, this statement compares the best achievable performance of each catalytic system and is not presented as evidence that the TiO2 polymorph alone determines defect density and CNT quality.

### 3.4. Surface Oxidation States and Electronic Metal–Support Interaction Probed by XPS

X-ray photoelectron spectroscopy (XPS) was used to examine the surface oxidation states and the interaction between TiO_2_ and the Fe–Co phase in n-anatase/Fe–Co/CNT, anatase/Fe–Co/CNT, and rutile/Fe–Co/CNT. Figure 6 shows the high-resolution C 1s, O 1s, Ti 2p, Co 2p_3_/_2_, and Fe 2p spectra, while the corresponding fitting parameters are summarized in Table S9. XRD analysis of the calcined catalysts previously identified crystalline TiO_2_ together with Co- and Fe-oxide phases, with no reflections attributable to metallic Fe or Co. XPS therefore complements the bulk structural analysis by resolving the surface oxidation states and electronic environments present after CNT growth.

The Ti 2p spectra show the characteristic Ti^4+^ 2p_3_/_2_–2p_1_/_2_ doublet for all three systems. The Ti^4+^ 2p_3_/_2_ component is located at 459.31, 459.07, and 459.11 eV for n-anatase, anatase, and rutile, respectively, while Ti^4+^ 2p_1_/_2_ appears at 465.01, 464.77, and 464.81 eV. The spin–orbit splitting remains approximately 5.70 eV. Relative to anatase and rutile, n-anatase exhibits positive Ti^4+^ 2p_3_/_2_ shifts of +0.24 and +0.20 eV, respectively, indicating a modified surface electronic environment.

An opposite trend is observed in the Fe 2p region. The Fe^0^ 2p_3_/_2_ component is centered at 706.98, 707.46, and 707.18 eV for n-anatase, anatase, and rutile, respectively; thus, n-anatase exhibits shifts of −0.48 and −0.20 eV relative to anatase and rutile. Its main oxidized Fe components are also shifted toward lower binding energy. The simultaneous positive shift in Ti^4+^ and negative shift in Fe is consistent with redistribution of electron density across the TiO_2_–Fe–Co interface and, therefore, with a support-dependent electronic metal–support interaction (EMSI). Son et al. [53] reported an analogous response for Pt/TiO_2_, where a negative Pt 4f shift accompanied by a positive Ti 2p shift was attributed to strong interfacial electronic coupling.

The comparison between n-anatase and anatase further relates this EMSI to support particle size. Decreasing the average particle diameter from 188.6 ± 71.4 to 30.2 ± 6.7 nm corresponds to an approximately 6.2-fold geometric increase in surface-to-volume ratio, assuming equivalent particle geometry and density. The higher surface-to-volume ratio increases the fraction of surface atoms and the potential Fe–Co/TiO_2_ interfacial area, while also changing surface curvature and local coordination. Thus, supports with the same anatase crystal phase can develop electronically distinct interfaces. Similarly, Zhao et al. [54] showed that the electronic properties of supported metals and their interaction with TiO_2_ depend on the TiO_2_ crystal structure, particle size, morphology, and stability.

The metallic contributions also differ among the three systems. Fe^0^ accounts for 11.15%, 10.92%, and 15.88%, whereas Co^0^ accounts for 12.03%, 12.36%, and 9.87% for n-anatase, anatase, and rutile, respectively. These results show different combinations of surface oxidation state and electronic environment for the Fe–Co phase. In a related Fe–Co system, Nánai et al. [55] used an AZO (Al-doped ZnO) substrate coated with Al_2_O_3_ and showed that the oxide support influences catalyst evolution and CNT growth during CCVD, supporting the general relevance of the oxide–FeCo interface during nanotube formation.

The C 1s spectra show very similar graphitic sp^2^ contributions for n-anatase and rutile (52.09% and 52.05%, respectively), whereas anatase shows a lower value of 46.35%. This trend agrees with the Raman results, where the corresponding (I_D_/I_G_) ratios are 0.59, 1.15, and 0.61 for n-anatase, anatase, and rutile, respectively. The low-binding-energy C 1s component near 283.9 eV remains essentially unchanged among the three systems, at 14.09%, 13.69%, and 13.78%, respectively. Li et al. [56] identified a Mo–C contribution at 283.5 eV in a Co–Mo_2_C/CNT system, supporting the tentative assignment of this low-binding-energy region to interfacial metal–carbon environments.

In the O 1s region, the lattice-oxygen contribution accounts for 38.36%, 30.02%, and 34.10% for n-anatase, anatase, and rutile, respectively. The higher-binding-energy components were considered collectively as surface oxygen species because contributions from hydroxyl groups and adsorbed oxygen-containing species may overlap in this region.

Overall, XPS complements the structural, morphological, and Raman characterization by revealing support-dependent differences in the surface oxidation states and electronic environment of the Fe–Co phase. These results provide the electronic basis for integrating the effects of TiO_2_ crystal phase, support particle size, and CNT growth conditions in the overall interpretation of the catalytic behavior.

## 4. Conclusions

This work reports a systematic, statistically designed evaluation of Fe–Co catalysts supported on three TiO_2_ systems for the synthesis of multi-walled carbon nanotubes by chemical vapor deposition. The conclusions are stated separately for the two comparisons supported by the experimental design.

Regarding the crystalline phase, the comparison between the two submicrometric supports shows that rutile and anatase achieve similar maximum carbon fixation yields (387.1% and 321.2%, respectively) but differ fundamentally in the shape of their operating window. The rutile-supported catalyst is essentially insensitive to temperature and growth time within the investigated range, providing yields between 336.9% and 387.2% across the entire range, while the anatase-supported catalyst varies by a factor of 2.2 within the same range and requires the upper corner of the design region to approximate the rutile’s performance.

Regarding support particle size, the comparison between submicrometric and nanocrystalline anatase (identical phase and calcination temperature) is the central result of this work. Reducing the support particle size from 188.6 ± 71.4 nm to 30.2 ± 6.7 nm raises the maximum carbon fixation yield from 321.2 ± 1.5% to 673.9 ± 31.4%. Under its best-performing growth condition, the nanocrystalline system also reduces the mean nanotube diameter from 48.4 ± 14.9 nm to 28.8 ± 10.5 nm, and lowers the I_D_/I_G_ ratio from 1.15 to 0.59; however, because these nanotubes were grown at 670 °C instead of 730 °C, these structural improvements reflect the combined effect of support particle size and growth temperature and cannot be attributed exclusively to the support. High carbon fixation yield is thus achieved without loss of structural quality. With respect to carbon fixation yield, the dominant variable in this study is thus the support particle size rather than the crystalline phase as such.

The statistical design also quantifies each system’s robustness. The n-anatase catalyst exceeds 400% carbon fixation yield in seven of the nine conditions tested and never falls below 334.6%, whereas neither of the other two systems reaches 400% under any condition. The best conditions identified for the two anatase systems lie at opposite temperature boundaries of the domain investigated and are reported as the best conditions within the range 670–730 °C and 30–60 min rather than as optima. For the nanocrystalline system, the fitted second-order model yields a saddle point rather than an interior maximum, consistent with the experimental maximum occurring at the lower temperature limit.

However, the present data do not allow the advantage of the nanocrystalline support to be ascribed solely to its smaller size. XPS analysis revealed a support-dependent metal–support electronic interaction; n-anatase exhibited the most pronounced Ti^4+^–Fe electronic perturbation, which is consistent with its distinctive catalytic behavior and superior performance in carbon fixation. This study proposes a hypothesis based on a larger surface area, a higher density of active sites, and improved dispersion of the Fe–Co phase within the nanocrystalline anatase. However, verifying this requires surface area measurements beyond the scope of this study.

In the future, it will be important to address other factors, as the scope of the conclusions is deliberately limited to a single carbon source (C_2_H_4_) and a single nominal Fe:Co ratio. Different proportions of hydrocarbons or metals could alter the order of the three supports, so it is important to address this aspect. It would also be interesting to examine a fourth catalyst supported on nanocrystalline rutile, with a particle size comparable to that of nanocrystalline anatase, calcined at the same temperature. This would allow for the separation of phase and size effects.

Overall, this work demonstrates that engineering the polymorphism and nanostructure of TiO_2_ supports is a highly effective strategy for improving the performance of Fe–Co catalysts in CNT synthesis. The combination of nanocrystalline anatase and optimized synthesis parameters enables the production of MWCNTs with high carbon fixation yield while maintaining good structural quality. These findings provide valuable insights for the rational design of advanced catalytic systems and contribute to the development of efficient routes for synthesizing carbon nanomaterials.

## Figures and Tables

**Figure 1:**
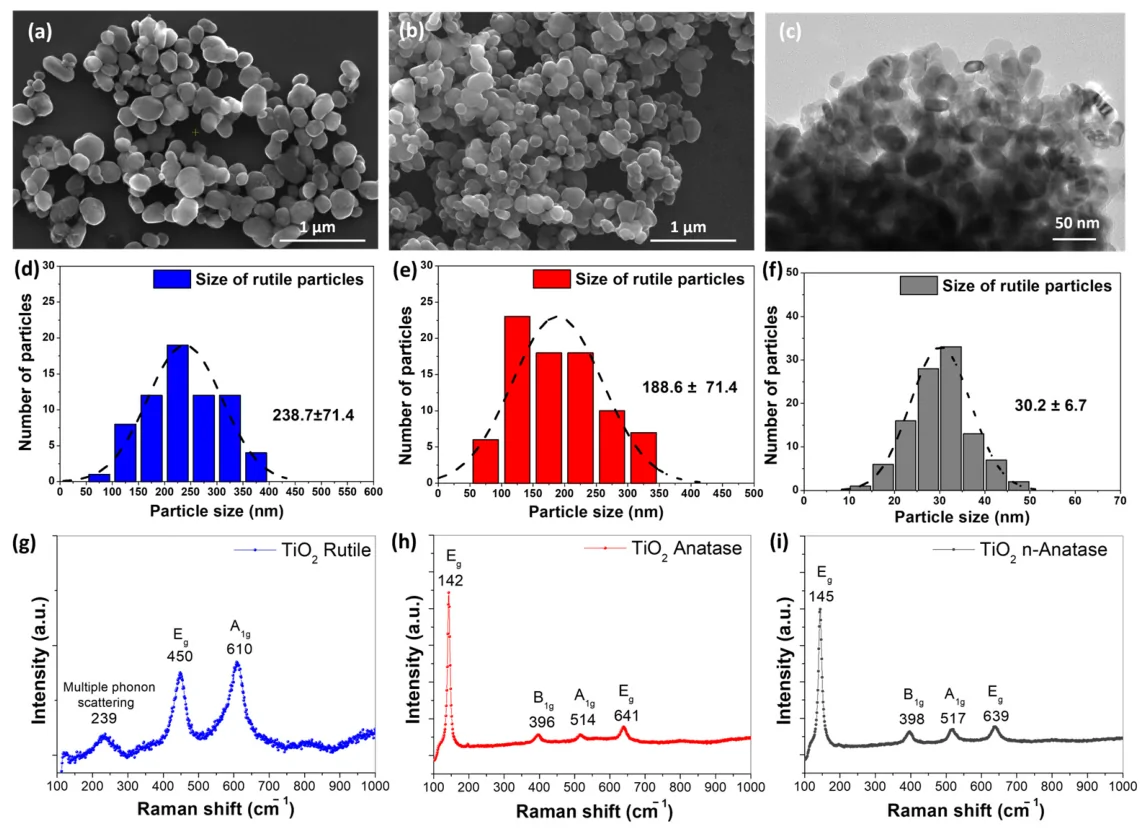
SEM images of (**a**) rutile particles, (**b**) anatase particles, (**c**) HRTEM of anatase nanoparticles. Particle-size histograms of (**d**) rutile, (**e**) anatase, (**f**) anatase nanoparticles. Raman spectra of (**g**) rutile particles, (**h**) anatase particles, and (**i**) anatase nanoparticles.

**Figure 2:**
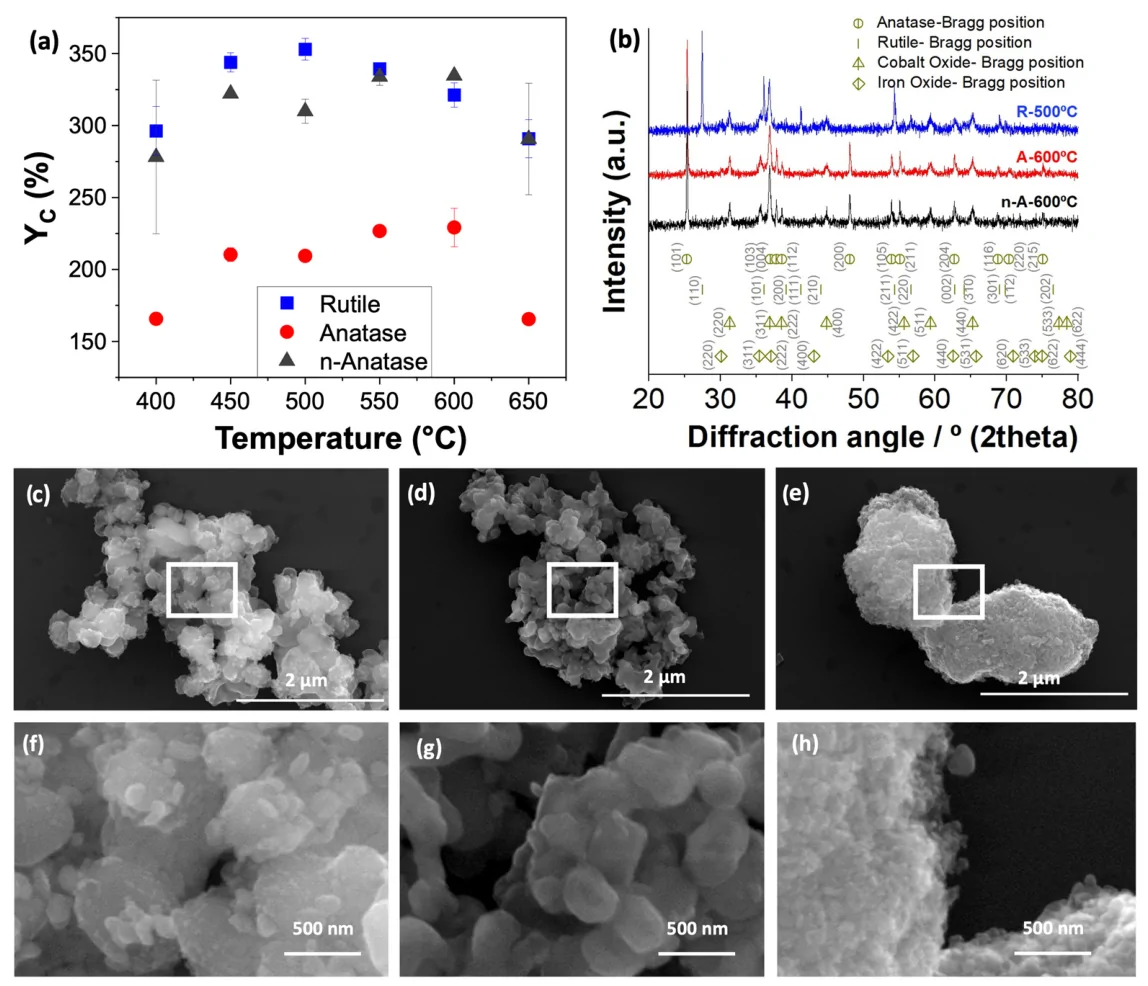
Results on the calcination temperature optimization of the TiO_2_/Fe–Co catalysts: (**a**) graph of carbon fixation yield as a function of calcination temperature of the TiO_2_/Fe–Co catalysts; time and calcination temperature were kept constant at 30 min and 730 °C, respectively. (**b**) XRD graph of the catalysts at the optimal calcination temperatures: in blue, the rutile/Fe–Co catalyst calcined at 500 °C; in red, the anatase/Fe–Co catalyst calcined at 600 °C; and in black, the n-anatase/Fe–Co catalyst calcined at 600 °C. FESEM images of the catalysts supported on: (**c**) rutile calcined at 500 °C; (**d**) anatase calcined at 600 °C; (**e**) n-anatase calcined at 600 °C; and (**f**–**h**) the magnified area of the rectangle in the center of each image, respectively.

**Figure 3:**
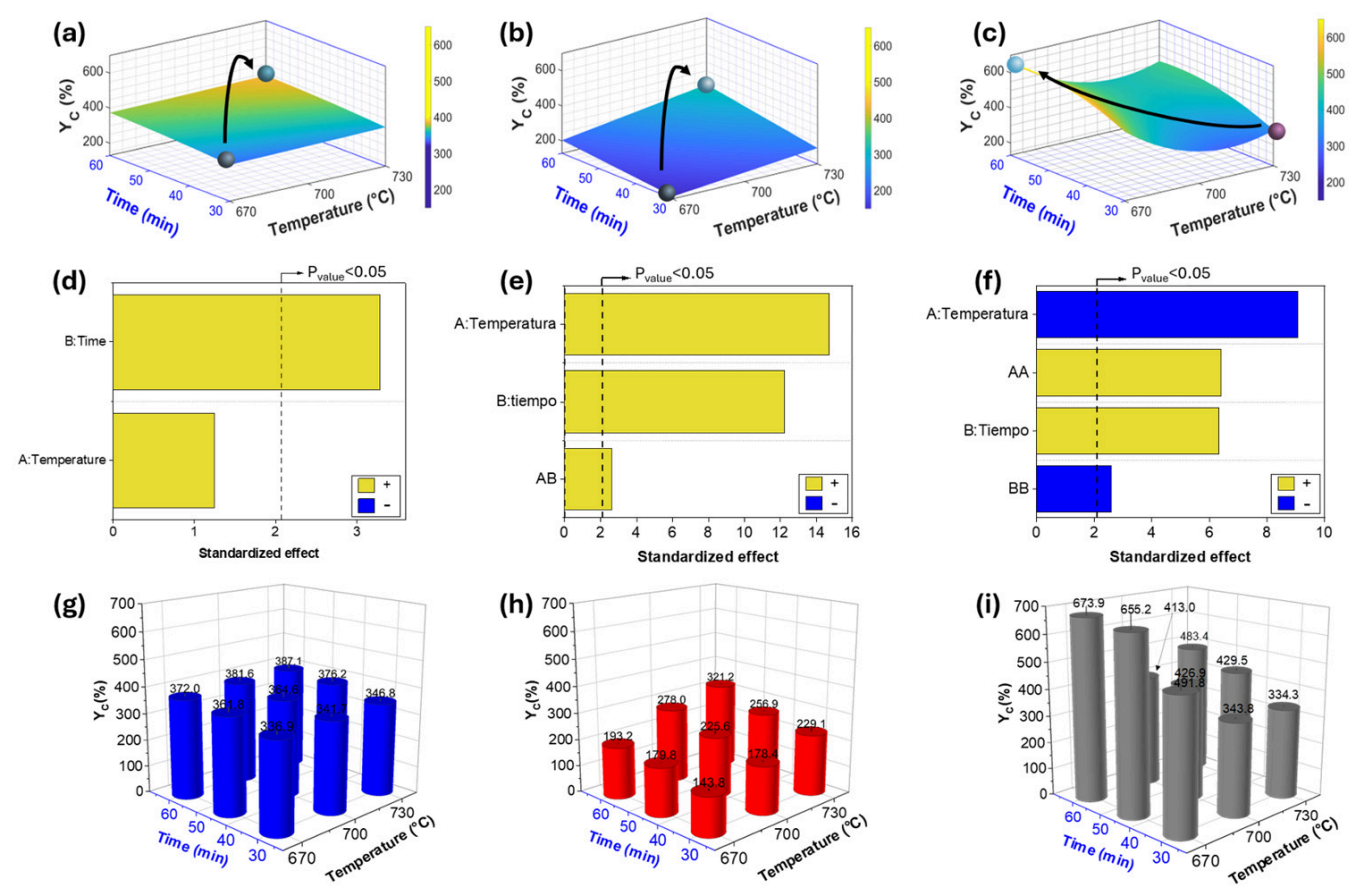
Analysis of the results of the optimization of CNT synthesis parameters to study carbon fixation yield using the three catalysts. Response surface of the design of experiments for carbon fixation yield as a function of time and temperature for: (**a**) Rutile/Fe–Co/CNT, (**b**) Anatase/Fe–Co/CNT, and (**c**) n-Anatase/Fe–Co/CNT. ANOVA analysis graphs for: (**d**) Rutile/Fe–Co/CNT, (**e**) Anatase/Fe–Co/CNT, and (**f**) n-Anatase/Fe–Co/CNT. Bar graphs of the carbon fixation yield obtained at different times and temperatures for each catalyst supported on (**g**) rutile, (**h**) anatase, and (**i**) n-anatase.

**Figure 4:**
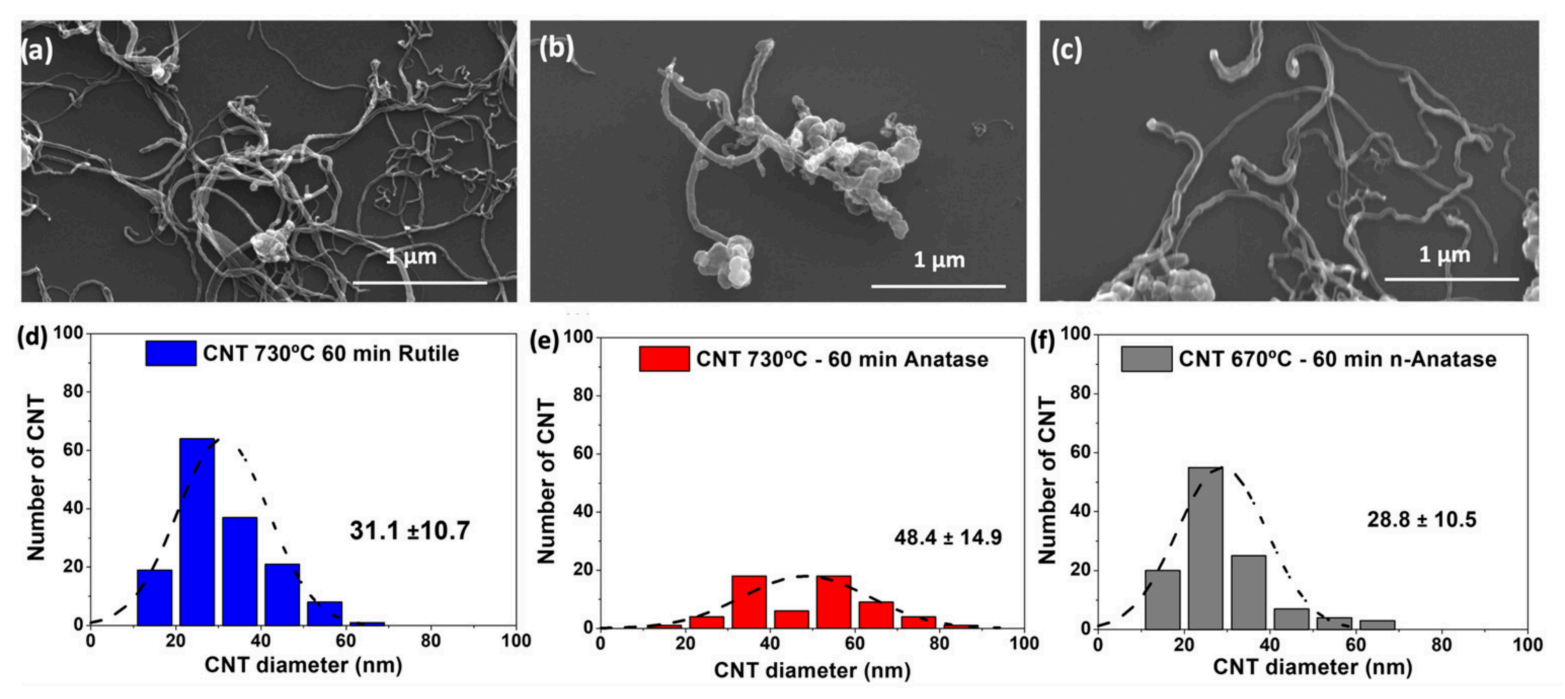
FESEM images of CNTs synthesized on catalyst (**a**) rutile/Fe–Co at 730 °C for 60 min, (**b**) anatase/Fe–Co at 730 °C for 60 min, (**c**) n-anatase/Fe–Co at 670 °C for 60 min. Histogram and statistics of the size of CNTs synthesized with catalyst (**d**) rutile/Fe–Co at 730 °C for 60 min, (**e**) anatase/Fe–Co at 730 °C for 60 min, (**f**) n-anatase/Fe–Co at 670 °C for 60 min.

**Figure 5:**
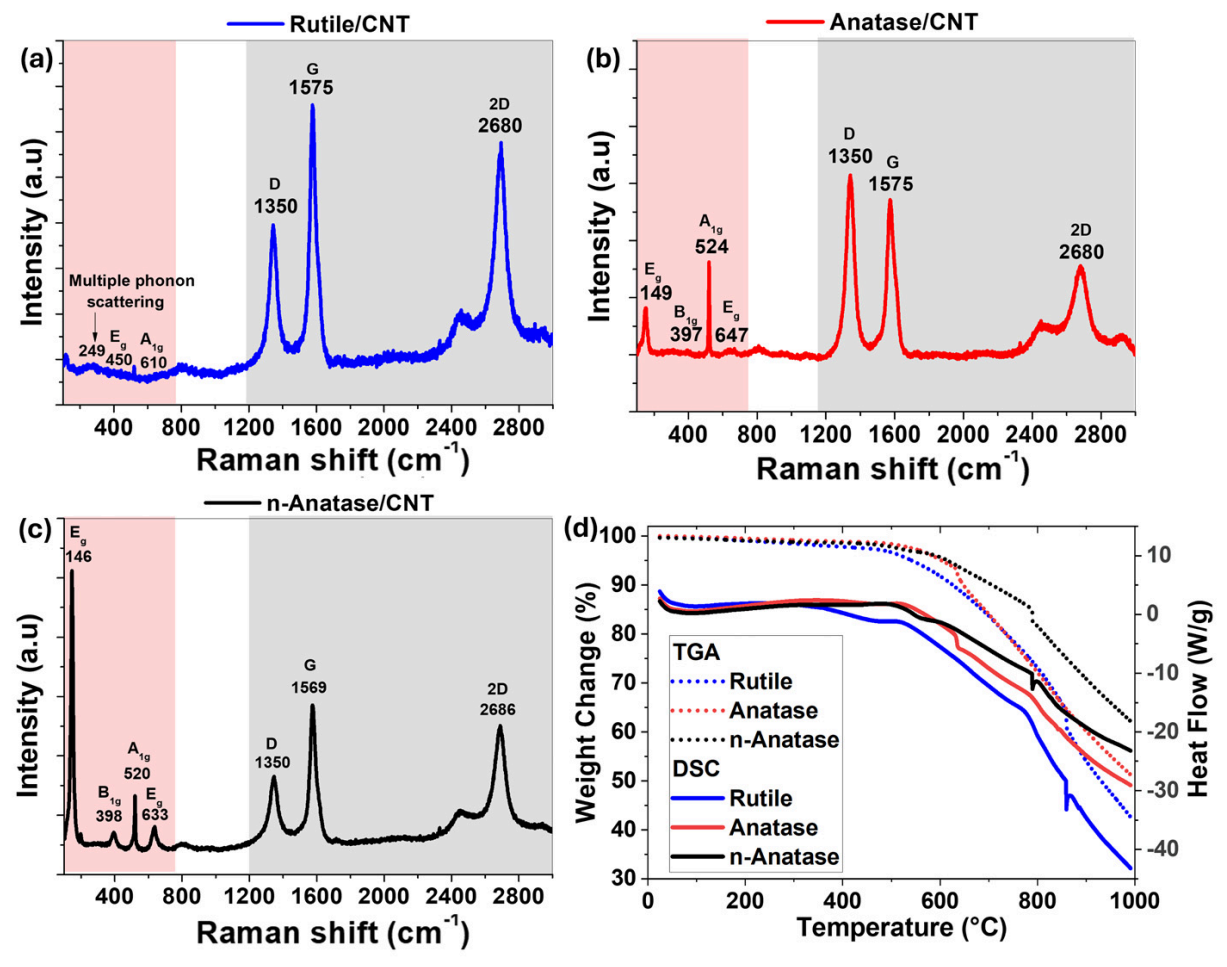
Raman spectroscopy graphs of CNTs synthesized on catalysts (**a**) rutile/Fe–Co at 730 °C for 60 min, (**b**) anatase/Fe–Co at 730 °C for 60 min, (**c**) n-anatase/Fe–Co at 670 °C for 60 min. The red (grey) background in Raman plots highlights the region where the TiO_2_ (CNT) spectra are observed. (**d**) TGA/DSC profiles of the CNTs synthesized on the three types of catalysts. The corresponding DTG curves are provided in Figure S5.

**Figure 6:**
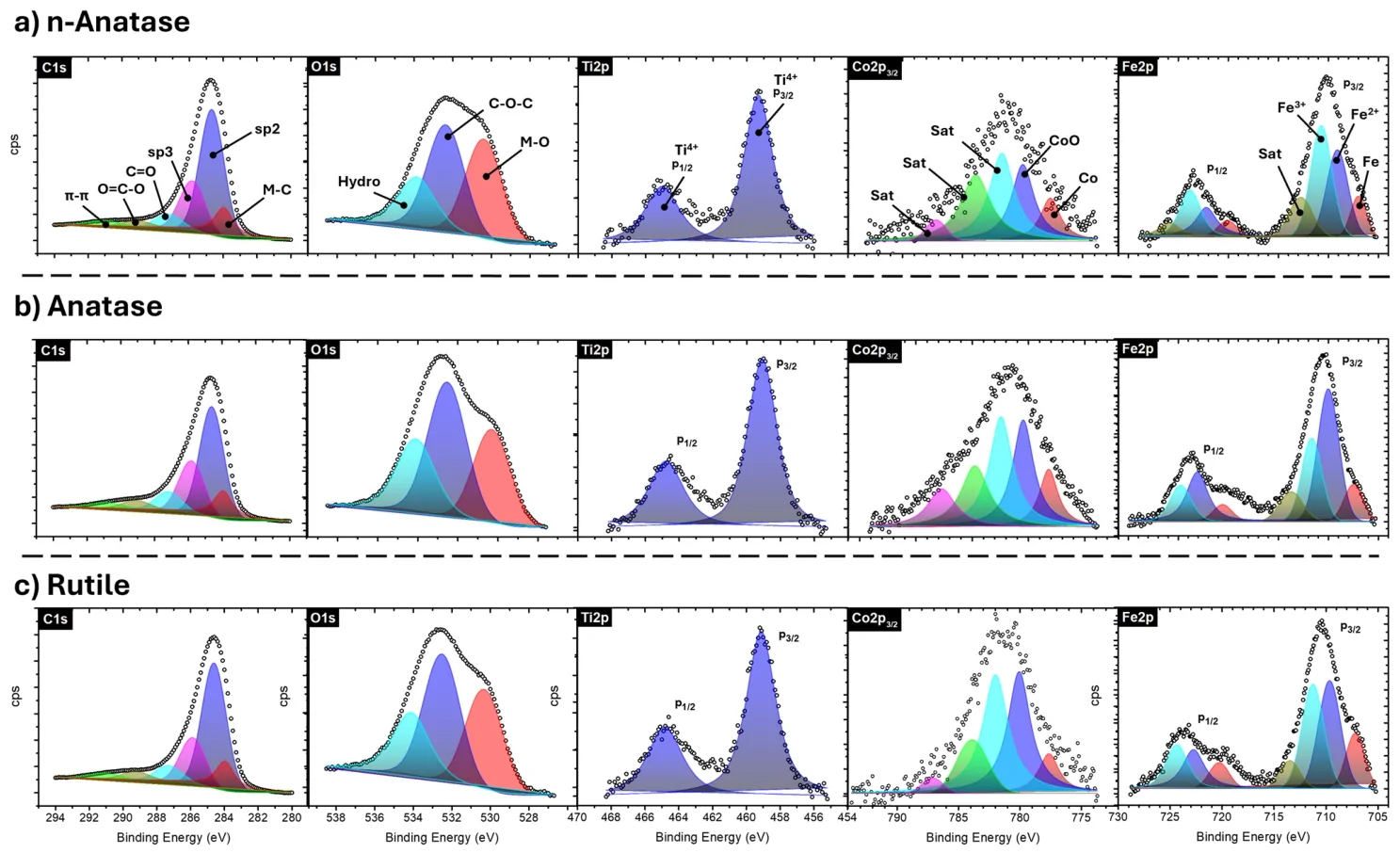
XPS spectra of CNTs synthesized on the catalysts: (**a**) n-anatase/Fe–Co at 670 °C for 60 min, (**b**) anatase/Fe–Co at 730 °C for 60 min, and (**c**) rutile/Fe–Co at 730 °C for 60 min.

**Table 1:** Comparison of MWCNT synthesis on TiO_2_-supported catalysts using CVD. For each system, the parameters governing the comparability of reported yields are listed: catalyst and support, carbon source, growth temperature and time, yield definition, and the denominator to which it refers.

Support/Catalyst	C Source	T (°C)	t (min)	(%) Yield Definition	Denominator	Reported Yield	Synthesis System	Ref.
n-anatase 5TiO_2_/2Fe_2_O_3_-6Co_3_O_4_	C_2_H_4_	670	60	(%) carbon fixation yield = [(mf − mi)/mi] × 100	calcined catalyst	673.9 ± 31.4%	CVD	This work
n-anatase 5TiO_2_/2Fe_2_O_3_-6Co_3_O_4_	C_2_H_4_	730	30	(%) carbon fixation yield = [(mf − mi)/mi] × 100	calcined catalyst	334.59 ± 2.99	CVD	This work
Anatase 5TiO_2_/2Fe_2_O_3_-6Co_3_O_4_	C_2_H_4_	730	30	(%) carbon fixation yield = [(mf − mi)/mi] × 100	calcined catalyst	229.13 ± 13.36	CVD	This work
Rutile 5TiO_2_/2Fe_2_O_3_-6Co_3_O_4_	C_2_H_4_	730	30	(%) carbon fixation yield = [(mf − mi)/mi] × 100	calcined catalyst	346.80 ± 8.17	CVD	This work
TiO_2_/5Co1Mo	Xylene	800	30	% of Carbon deposition = [(W2 − W1)/W1] × 100	Weight of catalyst powder	32.98% %	CCVD	[46]
TiO_2_/5Fe	Xylene	800	30	% of Carbon deposition = [(W2 − W1)/W1] × 100	Weight of catalyst powder	14.11%	CCVD	[46]
TiO_2_/5Co	Xylene	800	30	% of Carbon deposition = [(W2 − W1)/W1] × 100	Weight of catalyst powder	12.27%	CCVD	[46]
TiO_2_/5Fe1Mo	Xylene	800	30	% of Carbon deposition = [(W2 − W1)/W1] × 100	Weight of catalyst powder	16.01%	CCVD	[46]
TiO_2_/2.5Fe2.5Co	Xylene	800 °C	30	% of Carbon deposition = [(W2 − W1)/W1] × 100	Weight of catalyst powder	8.50%	CCVD	[46]
(rutile + anatase) Nanoparticles 1TiO_2_/10Fe(C_5_H_7_O_2_)_3_	C_2_H_2_	800	not reported	not reported	not reported	not reported	CVD	[51]

## Data Availability

The original contributions presented in this study are included in the article/Supplementary Material. Further inquiries can be directed to the corresponding authors.

## Supplementary Materials

The following supporting information can be downloaded at https://www.mdpi.com/article/10.3390/nano16191203/s1. Figure S1: X-ray diffraction patterns of the rutile/Fe–Co catalyst calcined in air for 4 h at 400–650 °C; Figure S2: X-ray diffraction patterns of the anatase/Fe–Co catalyst calcined in air for 4 h at 400–650 °C; Figure S3: X-ray diffraction patterns of the n-anatase/Fe–Co catalyst calcined in air for 4 h at 400–650 °C; Figure S4: Residual diagnostics for the fitted regression model; Figure S5: DSC/DTG analysis of CNTs synthesized on the three catalyst types; Table S1: Experimental matrix used for the optimization of the catalyst calcination temperature; Table S2: Factor levels of the 3 × 3 multilevel factorial design used for the optimization of the CNT synthesis parameters; Table S3: Carbon fixation yield obtained for CNT synthesis over the rutile/Fe–Co catalyst calcined at 500 °C; Table S4: Carbon fixation yield obtained for CNT synthesis over the anatase/Fe–Co catalyst calcined at 600 °C; Table S5: Carbon fixation yield obtained for CNT synthesis over the n-anatase/Fe–Co catalyst calcined at 600 °C; Table S6: Analysis of variance (ANOVA) of the carbon fixation yield for the rutile/Fe–Co catalytic system; Table S7: Analysis of variance (ANOVA) of the carbon fixation yield for the anatase/Fe–Co catalytic system; Table S8: Analysis of variance (ANOVA) of the carbon fixation yield for the n-anatase/Fe–Co catalytic system; Table S9: XPS peak-fitting parameters for the C 1s, O 1s, Ti 2p, Co 2p_3_/_2_, and Fe 2p regions of n-anatase/Fe–Co/CNT, anatase/Fe–Co/CNT, and rutile/Fe–Co/CNT.
