# Supplementary material for: A Systematic Study on High-Yield Carbon Nanotube Synthesis over TiO2-Supported Catalysts: Effect of TiO2 Polymorphism and Support Particle Size
Source: Nanomaterials (Basel). 2026 Sep 23;16(19):1203. doi: 10.3390/nano16191203 (PMC13667193; doi:10.3390/nano16191203)
Supplement: Supplementary file 1 [file nanomaterials-16-01203-s001.zip › nanomaterials-4534478-supplementary.pdf]

Supplementary Materials

A systematic study on high-yield carbon nanotube synthesis over TiO_2-supported catalysts: effect of TiO_2 polymorphism

Fernanda Olivares [1,*], Sergio Sauceda [2], Sheila Lascano[3], Cristina Arévalo[4], Rodrigo Segura[5], Marcos Flores[6] and Carolina Parra [3,*]

[1] Departamento de Física, Universidad Técnica Federico Santa María, Av. España 1680, Valparaíso, Chile; fernanda.olivaress@usm.cl

[2] Escuela de Ingeniería Mecánica, Pontificia Universidad Católica de Valparaíso, Los Carrera 01567, Quilpué, Chile; sergio.sauceda@pucv.cl

[3] Departamento de Ingeniería Mecánica, Universidad Técnica Federico Santa María, Santiago, Chile.; carolina.parra@usm.cl

[4] Departamento de Ingeniería y Ciencia de los Materiales y del Transporte, Escuela Politécnica Superior, Universidad de Sevilla, Calle Virgen de África 7, Sevilla 41011, España.; carevalo.us.es

[5] Instituto de Química, Universidad de Valparaíso, Gran Bretaña 1111, Valparaíso, Chile.; rodrigo.segura@uv.cl

[6] Department of Physics, Faculty of Physical and Mathematical Sciences, Universidad de Chile, Santiago 8370448, Chile; mflorescarra@uchile.cl

* Correspondence: carolina.parra@usm.cl, fernanda.olivaress@usm.cl

List of supporting information

Supplementary Figures

Figure S1. X-ray diffraction patterns of the rutile/Fe–Co catalyst calcined in air for 4 h at 400, 450, 500, 550, 600 and 650 °C.

Figure S2. X-ray diffraction patterns of the anatase/Fe–Co catalyst calcined in air for 4 h at 400, 450, 500, 550, 600 and 650 °C.

Figure S3. X-ray diffraction patterns of the n-anatase/Fe–Co catalyst calcined in air for 4 h at 400, 450, 500, 550, 600 and 650 °C.

Figure S4. Residual diagnostics for the fitted regression models.

Figure S5. DSC/DTG analysis of CNTs synthesized on the three catalyst types.

Supplementary Tables

Table S1. Experimental matrix used for the optimization of the catalyst calcination temperature.

Table S2. Factor levels of the 3 × 3 multilevel factorial design used for the optimization of the CNT synthesis parameters.

Table S3. Carbon fixation yield obtained for CNT synthesis over the rutile/Fe–Co catalyst calcined at 500 °C.

Table S4. Carbon fixation yield obtained for CNT synthesis over the anatase/Fe–Co catalyst calcined at 600 °C.

Table S5. Carbon fixation yield obtained for CNT synthesis over the n-anatase/Fe–Co catalyst calcined at 600 °C.

Table S6. Analysis of variance (ANOVA) of the carbon fixation yield for the rutile/Fe–Co catalytic system.

Table S7. Analysis of variance (ANOVA) of the carbon fixation yield for the anatase/Fe–Co catalytic system.

Table S8. Analysis of variance (ANOVA) of the carbon fixation yield for the n-anatase/Fe–Co catalytic system.

Table S9. XPS peak-fitting parameters for the C 1s, O 1s, Ti 2p, Co $2p_{3/2}$, and Fe 2p regions of n-anatase/Fe–Co/CNT, anatase/Fe–Co/CNT, and rutile/Fe–Co/CNT.

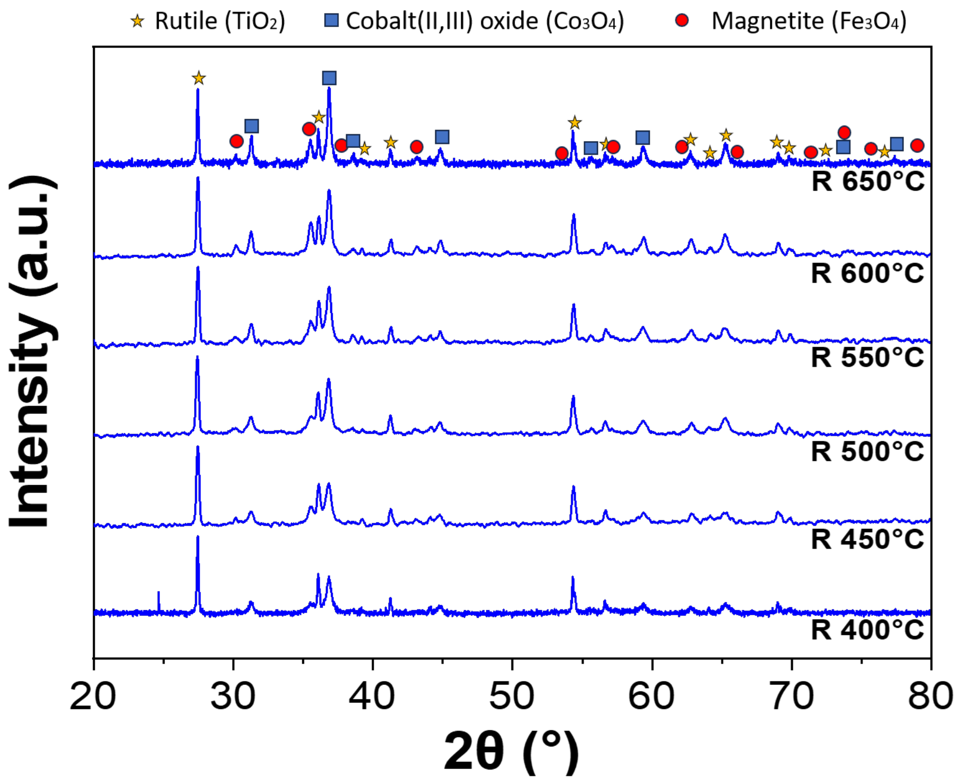

Figure S1. X-ray diffraction patterns of the rutile/Fe–Co catalyst calcined in air for 4 h at 400, 450, 500, 550, 600, and 650 °C. The reflections of tetragonal rutile TiO_2 (PDF 00-021-1276) are retained over the whole temperature interval, confirming that the support does not undergo any phase transformation and that the effect of the calcination temperature is restricted to the supported Fe–Co oxide phases. The reflections assigned to cubic Co_3O_4 (PDF 00-042-1467) and iron oxide (PDF 04-015-9120) become progressively more intense and better defined as the calcination temperature increases, indicating crystallization and subsequent coarsening of the oxide precursors of the active phase.

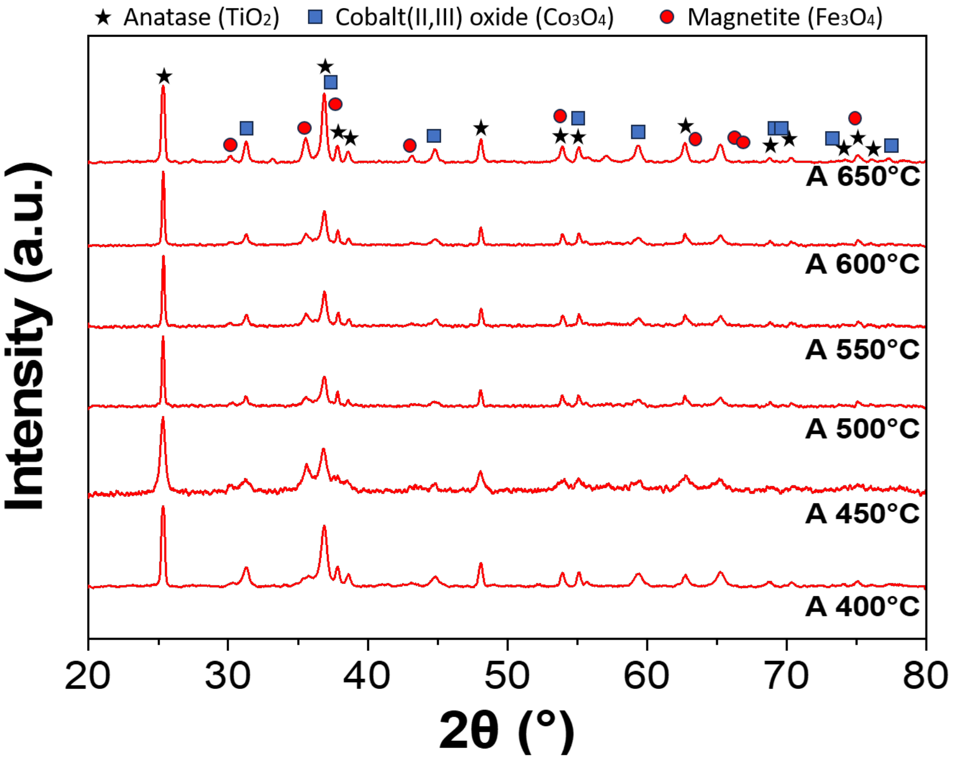

Figure S2. X-ray diffraction patterns of the anatase/Fe–Co catalyst calcined in air for 4 h at 400, 450, 500, 550, 600 and 650 °C. The reflections of tetragonal anatase TiO_2 (PDF 00-021-1272) are preserved throughout the series, indicating that the calcination window explored lies below the anatase-to-rutile transformation threshold. As for the rutile-supported system, the Co_3O_4 (PDF 00-042-1467) and iron oxide (PDF 04-015-9120) reflections sharpen with increasing calcination temperature.

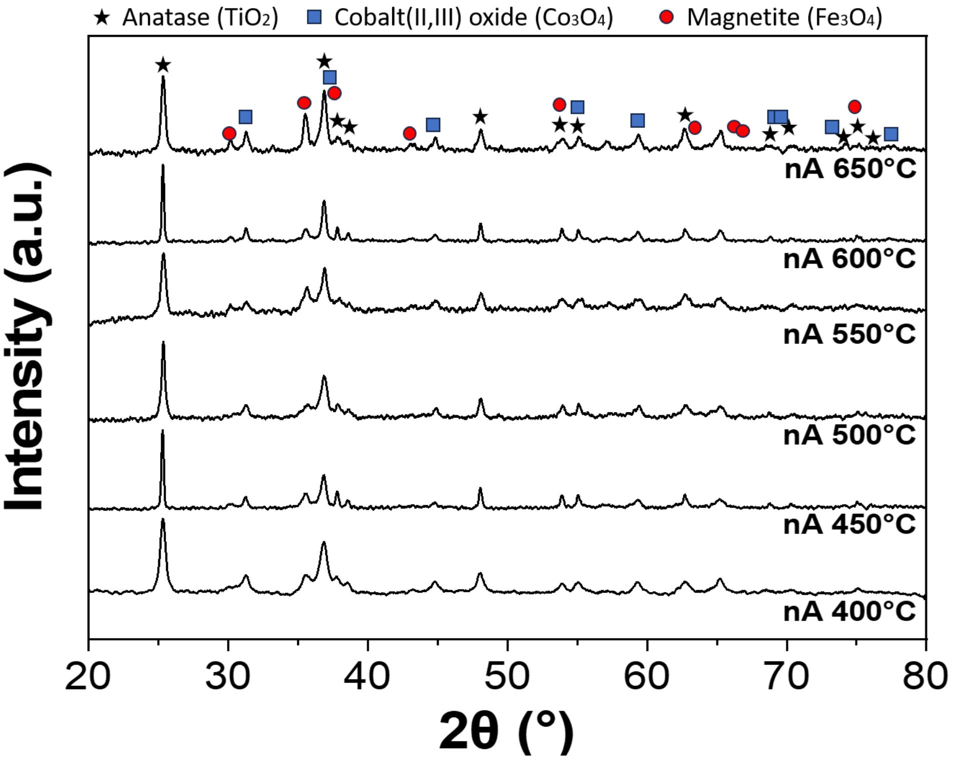

Figure S3. X-ray diffraction patterns of the n-anatase/Fe–Co catalyst calcined in air for 4 h at 400, 450, 500, 550, 600 and 650 °C. The anatase framework (PDF 00-021-1272) is retained over the entire interval. The reflections are markedly broader than those of the micrometric anatase-supported catalyst (Figure S2), consistent with the nanometric crystallite size of the support determined by microscopy (30.2 ± 6.7 nm), and no additional crystalline phases are detected besides Co_3O_4 (PDF 00-042-1467) and iron oxide (PDF 04-015-9120).

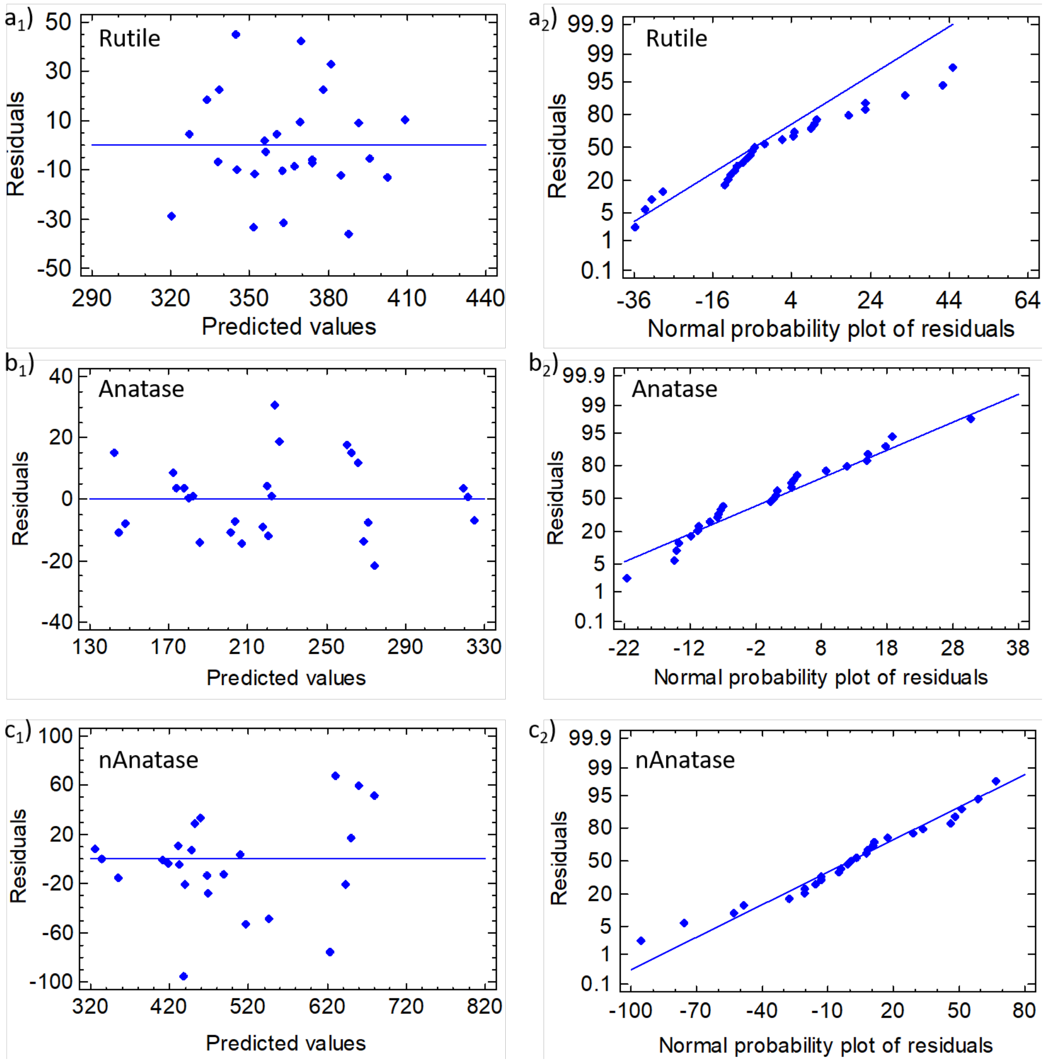

Figure S4. Residual diagnostic plots for the regression models fitted to the carbon fixation yield of the (a) Rutile/Fe–Co/CNT, (b) Anatase/Fe–Co/CNT, and (c) n-Anatase/Fe–Co/CNT catalytic systems. Panels a1–c1 show residuals versus predicted values, with no clear systematic trend or funnel-shaped dispersion. Panels a2–c2 show the normal probability plots of the residuals; Anatase and n-Anatase follow the reference line closely, while the Rutile model shows a slight departure from linearity, consistent with the inter-block variability observed in this system.

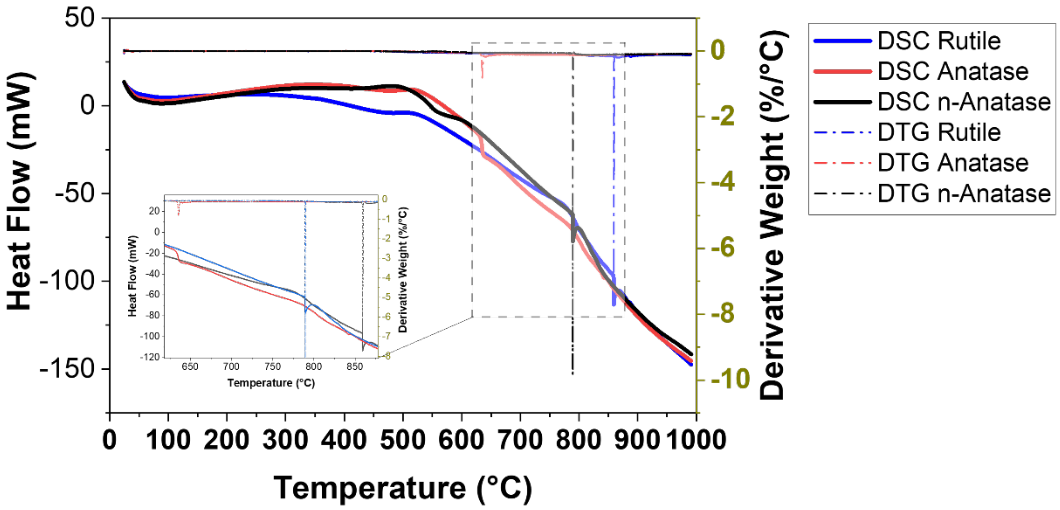

Figure S5. DSC/DTG analysis of the CNTs synthesized over the three catalyst systems shows sharp signal changes at approximately 860 °C, 635 °C, and 790 °C for Rutile, Anatase, and n-Anatase, respectively. Because these localized features coincide with abrupt discontinuities in both the DTG and heat-flow signals, they were not used to define characteristic oxidation temperatures.

Table S1. Experimental matrix used for the optimization of the catalyst calcination temperature. For each TiO_2 support (rutile, anatase and nanocrystalline anatase), six calcination temperatures between 400 and 650 °C (4 h, static air) were evaluated, while the CNT synthesis temperature and residence time were held constant at 730 °C and 30 min, respectively, so that any variation in the carbon fixation yield can be attributed exclusively to the thermal history of the catalyst.

Catalyst						
**Synthesis T (°C)**	**Time (min)**	**Catalyst**	**Calcination T (°C)**	**C Yield (%)**	**standard deviation (%)**	**Error (%)**
730	30	submicrometer rutile	400	296.26	29.49	17.03
			450	343.91	11.51	6.64
			500	353.00	13.05	7.54
			550	339.39	7.59	4.38
			600	321.21	14.63	8.45
			650	290.90	23.00	13.28
		nanometric anatase	400	278.16	92.32	53.30
			450	210.31	8.30	4.79
			500	309.92	14.50	8.37
			550	333.88	10.26	5.92
			600	334.59	5.19	2.99
			650	290.64	67.14	38.76
		submicrometer anatase	400	165.68	4.99	2.88
			450	322.17	2.57	1.49
			500	209.41	7.38	4.26
			550	226.68	1.37	0.79
			600	229.13	23.14	13.36
			650	165.50	4.42	2.55

Table S2. Factor levels of the 3 × 3 multilevel factorial design used for the optimization of the CNT synthesis parameters. For each catalyst, the calcination temperature was fixed at the optimal value identified in the first experimental design (500 °C for rutile/Fe–Co; 600 °C for anatase/Fe–Co and n-anatase/Fe–Co), and the synthesis temperature (670, 700 and 730 °C) and residence time (30, 45 and 60 min) were varied in a completely randomized design with three replicates per condition (27 runs per catalyst).

**CNT**								
**Rutile**			**Anatase**			**n-Anatase**		
**T° (C) Cal**	**T(°C)**	**t (min)**	**T° (C) Cal**	**T(°C)**	**t (min)**	**T° (C) Cal**	**T(°C)**	**t (min)**
500	670	30	600	670	30	600	670	30
		45			45			45
		60			60			60
	700	30		700	30		700	30
		45			45			45
		60			60			60
	730	30		730	30		730	30
		45			45			45
		60			60			60

Table S3. Carbon fixation yield obtained for CNT synthesis over the rutile/Fe–Co catalyst calcined at 500 °C. For each temperature–time combination of the factorial design, the initial catalyst mass, the mass recovered after growth and the resulting carbon fixation yield (Y_C) are reported as mean ± standard deviation of three independent replicates.

CNT synthesis					
**T° (C) Cal**	**T(°C)**	**t (min)**	**catalyst mass (g)**	**C mass (g)**	**Y_C (%)**
500	670	30	0.1009 ± 0.0003	0.4408 ± 0.0178	336.91 ± 17.04
		45	0.1007 ± 0.0004	0.4653 ± 0.0228	361.84 ± 20.42
		60	0.1008 ± 0.0002	0.4758 ± 0.0105	372.03 ± 9.67
	700	30	0.1006 ± 0.0001	0.4445 ± 0.0086	341.70 ± 8.46
		45	0.1007 ± 0.0003	0.4680 ± 0.0188	364.61 ± 17.27
		60	0.1008 ± 0.0001	0.4853 ± 0.0173	381.62 ± 17.80
	730	30	0.1015 ± 0.0007	0.4535 ± 0.0115	346.80 ± 7.5351
		45	0.1012 ± 0.0001	0.4824 ± 0.0211	376.18 ± 20.92
		60	0.1012 ± 0.0002	0.4931 ± 0.0188	387.14 ± 19.68

Table S4. Carbon fixation yield obtained for CNT synthesis over the anatase/Fe–Co catalyst calcined at 600 °C. For each temperature–time combination of the factorial design, the initial catalyst mass, the mass recovered after growth and the resulting carbon fixation yield (Y_C) are reported as mean ± standard deviation of three independent replicates.

CNT synthesis					
**T° (C) Cal**	**T(°C)**	**t (min)**	**catalyst mass (g)**	**C mass (g)**	**Y_C (%)**
600	670	30	0.1003 ± 0.0001	0.2444 ± 0.0069	143.76 ± 7.11
		45	0.1007 ± 0.0001	0.2816 ± 0.0015	179.77 ± 1.05
		60	0.1009 ± 0.0001	0.2958 ± 0.0018	193.22 ± 1.57
	700	30	0.1005 ± 0.0001	0.2797 ± 0.0037	178.39 ± 3.46
		45	0.1008 ± 0.0001	0.3282 ± 0.0110	225.65 ± 10.56
		60	0.1005 ± 0.0001	0.3800 ± 0.0004	277.98 ± 0.23
	730	30	0.1002 ± 0.0001	0.3297 ± 0.0132	229.13 ± 13.36
		45	0.1009 ± 0.0001	0.3600 ± 0.0032	256.91 ± 3.18
		60	0.1006 ± 0.0002	0.4237 ± 0.0020	321.17 ± 1.49

Table S5. Carbon fixation yield obtained for CNT synthesis over the n-anatase/Fe–Co catalyst calcined at 600 °C. For each temperature–time combination of the factorial design, the initial catalyst mass, the mass recovered after growth and the resulting carbon fixation yield (Y_C) are reported as mean ± standard deviation of three independent replicates.

CNT synthesis					
**T° (C) Cal**	**T(°C)**	**t (min)**	**catalyst mass (g)**	**C mass (g)**	**Y_C (%)**
600	670	30	0.1004 ± 0.0003	0.5941± 0.0162	491.82 ± 14.55
		45	0.1003 ± 0.0001	0.7576± 0.0532	655.19 ± 53.89
		60	0.1004 ± 0.0002	0.7765± 0.0303	673.87 ± 31.37
	700	30	0.1004 ± 0.0002	0.4454± 0.0047	343.78 ± 4.85
		45	0.1009 ± 0.0003	0.5317± 0.0132	426.88 ± 14.32
		60	0.1008 ± 0.0002	0.5170± 0.0359	413.02 ± 35.37
	730	30	0.1003 ± 0.0003	0.4357± 0.0022	334.59 ± 2.99
		45	0.1009 ± 0.0003	0.5344± 0.0071	429.48 ± 6.46
		60	0.1004 ± 0.0003	0.5857± 0.0043	483.39 ± 5.08

Table S6. Analysis of variance (ANOVA) of the carbon fixation yield for the rutile/Fe–Co catalytic system, evaluating the effect of the synthesis temperature (A), the residence time (B) and the experimental block. Only the residence time is significant at the 95% confidence level ($p = 0.0034$); the synthesis temperature does not reach significance ($p = 0.2261$) and the block effect is significant ($p = 0.0050$), which together with the low adjusted R^2 (45.7%) reflects the flatness of the response surface of this catalyst.

**Factors**	**SS**	**DF**	**MS**	**F**	$\mathbf{P_{value}}$
A:Temperature	825.398	1	825.398	1.55	0.2261
B:Time	5726.28	1	5726.28	10.76	0.0034
blocks	7230.89	2	3615.44	6.79	0.005
Error	11709.1	22	532.232		
Total	25491.7	26			
R^2	54.1%				
$R^2_{Adjusted}$	45.7%				

SS: sequential sum of squares, DF: degree of freedom, MS: mean square, F: statistical test

Table S7. Analysis of variance (ANOVA) of the carbon fixation yield for the anatase/Fe–Co catalytic system. Both main effects, the synthesis temperature (A) and the residence time (B), are highly significant ($p < 0.0001$), as is their two-factor interaction (AB, $p = 0.0153$), whereas the block effect is not significant ($p = 0.6836$). The adjusted R^2 of 93.4% confirms the adequacy of the model.

**Factors**	**SS**	**DF**	**MS**	**F**	**P_{value}**
A:Temperature	42182.5	1	42182.5	216.51	0
B:Time	29061.4	1	29061.4	149.16	0
AB	1359.58	1	1359.58	6.98	0.0153
blocks	150.967	2	75.4835	0.39	0.6836
Error	4091.47	21	194.832		
Total	76845.9	26			
R^2	94.7%				
$R^2_{Adjusted}$	93.4%				

SS: sequential sum of squares, DF: degree of freedom, MS: mean square, F: statistical test

Table S8. Analysis of variance (ANOVA) of the carbon fixation yield for the n-anatase/Fe–Co catalytic system. In addition to the two main effects, the synthesis temperature (A) and the residence time (B), the quadratic terms AA ($p < 0.0001$) and BB ($p = 0.0178$) are significant, indicating a genuinely curved response surface with an interior optimum; the block effect is not significant ($p = 0.2102$) and the adjusted R^2 is 86.6%.

**Factors**	**SS**	**DF**	**MS**	**F**	**P_{value}**
A:Temperatura	164411	1	164411	82.28	0
B:Tiempo	80036	1	80036	40.05	0
AA	81894.7	1	81894.7	40.98	0
BB	13312.7	1	13312.7	6.66	0.0178
bloques	6744.92	2	3372.46	1.69	0.2102
Error	39965	20	1998.25		
Total	386364	26			
R^2	89.7%				
$R^2_{Adjusted}$	86.6%				

SS: sequential sum of squares, DF: degree of freedom, MS: mean square, F: statistical test

Table S9. XPS peak-fitting parameters for the C 1s, O 1s, Ti 2p, Co $2p_{3/2}$, and Fe 2p regions of n-anatase/Fe–Co/CNT, anatase/Fe–Co/CNT, and rutile/Fe–Co/CNT.

Sample	n-Anatase	Anatase	Rutile
Ti^{4+} $2p_{3/2}$ BE (eV)	459,31	459,07	459,11
ΔBE Ti vs Rutile (eV)	0,2	-0,05	0
Fe^0 $2p_{3/2}$ BE (eV)	706,98	707,46	707,18
ΔBE Fe^0 vs Rutile (eV)	-0,2	0,28	0
Apparent Fe0 (%)	11,15	10,92	15,88
Co^0 $2p_{3/2}$ BE (eV)	777,67	778,19	777,63
ΔBE Co^0 vs Rutile (eV)	0,04	0,56	0
Apparent Co^0 (%)	12,03	12,36	9,87
Co^0 among main Co states (%)	17,75	18,11	12,02
C sp^2 (%)	52,09	46,35	52,05
Low-BE C-M (%)	14,09	13,69	13,78
Lattice O (%)	38,36	30,02	34,1
Non-lattice O (%)	61,64	69,98	65,9
